# Multiomic analysis of familial adenomatous polyposis reveals molecular pathways associated with early tumorigenesis

**DOI:** 10.1038/s43018-024-00831-z

**Published:** 2024-10-30

**Authors:** Edward D. Esplin, Casey Hanson, Si Wu, Aaron M. Horning, Nasim Bararpour, Stephanie A. Nevins, Lihua Jiang, Kévin Contrepois, Hayan Lee, Tuhin K. Guha, Zheng Hu, Rozelle Laquindanum, Meredith A. Mills, Hassan Chaib, Roxanne Chiu, Ruiqi Jian, Joanne Chan, Mathew Ellenberger, Winston R. Becker, Bahareh Bahmani, Aziz Khan, Basil Michael, Annika K. Weimer, D. Glen Esplin, Jeanne Shen, Samuel Lancaster, Emma Monte, Thomas V. Karathanos, Uri Ladabaum, Teri A. Longacre, Anshul Kundaje, Christina Curtis, William J. Greenleaf, James M. Ford, Michael P. Snyder

**Affiliations:** 1 Department of Genetics, Stanford School of Medicine, Stanford, CA USA; 2 Department of Medicine, Stanford School of Medicine, Stanford, CA USA; 3 Stanford Cancer Institute, Stanford School of Medicine, Stanford, CA USA; 4 Novo Nordisk Foundation Center for Genomic Mechanisms of Disease, Broad Institute of MIT and Harvard, Cambridge, MA USA; 5 Animal Reference Pathology, Salt Lake City, UT USA; 6 Department of Pathology, Stanford School of Medicine, Stanford, CA USA; 7 Department of Computer Science, Stanford University, Stanford, CA USA; 8 Chan Zuckerberg Biohub, San Francisco, CA USA

**Keywords:** Colon cancer, Cancer, Computational biology and bioinformatics

## Abstract

Familial adenomatous polyposis (FAP) is a genetic disease causing hundreds of premalignant polyps in affected persons and is an ideal model to study transitions of early precancer states to colorectal cancer (CRC). We performed deep multiomic profiling of 93 samples, including normal mucosa, benign polyps and dysplastic polyps, from six persons with FAP. Transcriptomic, proteomic, metabolomic and lipidomic analyses revealed a dynamic choreography of thousands of molecular and cellular events that occur during precancerous transitions toward cancer formation. These involve processes such as cell proliferation, immune response, metabolic alterations (including amino acids and lipids), hormones and extracellular matrix proteins. Interestingly, activation of the arachidonic acid pathway was found to occur early in hyperplasia; this pathway is targeted by aspirin and other nonsteroidal anti-inflammatory drugs, a preventative treatment under investigation in persons with FAP. Overall, our results reveal key genomic, cellular and molecular events during the earliest steps in CRC formation and potential mechanisms of pharmaceutical prophylaxis.

## Abstract (web summary)

Snyder and colleagues present a comprehensive multiomic atlas of normal mucosal, benign polyps and dysplastic polyps from six persons with familial adenomatous polyposis, comprising transcriptomic, proteomic, metabolomic and lipidomic datasets.

## Main

Colorectal adenocarcinoma (CRC) is the third leading cause of cancer death in the United States, with more than 140,000 new cases each year; thus, it is a major public health burden^1^. Understanding the molecular pathways involved in the genesis of both sporadic and hereditary CRCs is critical for improved diagnosis, as well as for developing preventive and therapeutic interventions^2^. The earliest molecular and biochemical alterations involved in CRC tumorigenesis remain poorly understood as precursor lesions have been minimally studied, as has the surrounding mucosa and dysplastic tissue.

Familial adenomatous polyposis (FAP) is an ideal system in which to study early events during CRC formation for multiple reasons. First, more than 95% of persons with FAP harbor germline mutations in *APC*, a key component of the Wnt signaling pathway that is somatically altered in 93% of all CRCs and presumed to be the initiating event^3,4^. Second, persons with FAP develop hundreds to thousands of polyps in the colon, often starting in adolescence where ‘small’ (<0.5 mm) polyps appear benign and those >0.5 mm to 1 cm usually exhibit dysplasia. Third, persons with FAP have a nearly 100% lifetime risk of developing invasive adenocarcinomas and, thus, often have their entire colon removed as prophylactic treatment once the number of polyps that develop becomes unmanageable through endoscopic surveillance. Accordingly, molecular analysis of multiple benign and dysplastic polyps from the same person, as well as ‘normal mucosa’ can reveal the mechanisms by which *APC* mutations initiate premalignant polyps and their transition to a malignant state across a common germline genetic background. Such information is expected to enhance our understanding of the requisite alterations for transformation in both hereditary and sporadic CRC and may inform the approach to prevention, early detection and treatment^5,6^.

To better understand events that occur during CRC formation and as part of the Human Tumor Atlas Network (HTAN)^7^, we performed transcriptome, proteome, metabolome and lipidome analyses of *n* = 93 samples from six persons with FAP (5–28 per person) representing different stages of cancer progression: unaffected colonic mucosa, benign polyps and dysplastic polyps. These data delineate cellular and molecular changes during early tumorigenesis through the transition to invasive disease, providing insights into the mechanisms of CRC progression and its prevention. This rich open-access multiomic, multisample dataset also provides a valuable scientific resource to the community, complementing the existing datasets on sporadic CRCs.

## Results

### Multiomic analysis of FAP samples

**Fig. 1:**
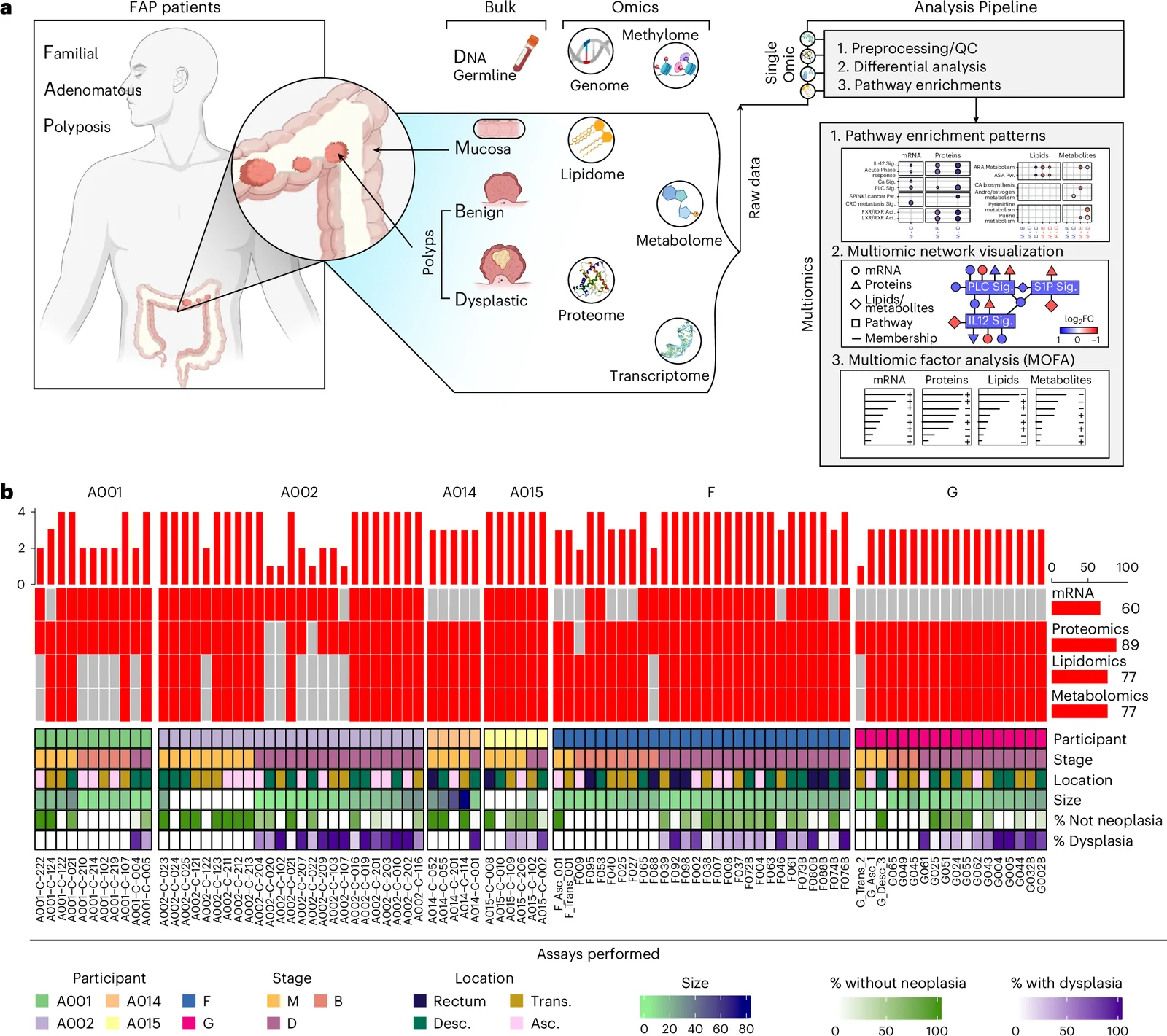
Overview of the multiomic FAP study. **a**, Schematic of tissue and blood collections, assays and downstream single-omic and multiomic analyses performed in this study. **b**, Summary of the clinical features of each of 93 colorectal samples derived from six persons with FAP, with up to four assays were performed on each sample. Portions of this figure were created with BioRender.com. Source data

We collected and analyzed *n* = 93 samples from the colons of six persons with FAP whose characteristics are shown in Table 1. The samples were isolated from multiple regions of the colon: ascending, transverse, descending (including sigmoid) and rectum (Fig. 1a). Samples spanned a range of sizes and degrees of dysplasia determined using histological staining: 26 polyp-adjacent ‘normal’ mucosa samples (hereafter referred to in the text as ‘mucosa’), 16 benign polyps (usually small) and 51 dysplastic polyps (small to large). The tumor percentage and degree of dysplasia in the dysplastic polyps were scored by a trained pathologist.

**Table 1:** Demographic and clinical characteristics of participants in the cohort

Identifier	Age at colectomy (sample collection)	Sex	Race (ethnicity)	Syndrome	Age of diagnosis	Age of genetic test	Positive gene	Gene site
A001	45	Male	Hispanic/Latino	FAP	Unknown	None	Unknown	Unknown—clinical diagnosis and family history
A002	20	Female	White	FAP	18	18	*APC*	c.3183_3187delACAAA (p.Q1062*)
A014	22	Female	White	FAP	21	21	Negative	Negative
A015	35	Female	Hispanic/Latino	FAP	35	35	*APC*	c.667C>T (p.Q223*)
F	24	Female	Hispanic/Latino	FAP	23	23	*APC*	Q541X (c.1621C>T)
G	28	Male	Hispanic/Latino	FAP	27	27	*APC*	Q541X (c.1621C>T)

Samples were subjected to a battery of multiomic assays including bulk RNA sequencing (RNA-seq), proteomics (tandem mass tag (TMT) labeling), untargeted metabolomics (reverse-phase liquid chromatography (RPLC) and hydrophilic interaction LC (HILIC) combined with mass spectrometry (MS)^8^) and targeted lipidomics (Fig. 1b). High-resolution single-cell (sc) assays, including single-nucleus (sn) assays for RNA-seq and assay for transposase-accessible chromatin with sequencing (ATAC-seq) were performed (described in a recent manuscript^9^), along with methylation analyses^10^.

### Extensive molecular changes in polyp development

To understand the molecular pathways activated during the early stages of polyp formation and cancer progression, we performed deep multiomic profiling of histologically normal mucosa, benign polyps and dysplastic polyps (Fig. 1a). RNA-seq, TMT-based proteomics, untargeted metabolomics and targeted lipidomics were performed on 60, 89, 77 and 77 samples, respectively. Because of limited material, particularly for the small polyps, not all assays could be performed for all samples. The relative levels of transcripts (*n* = 23,512), proteins (*n* = 12,389), metabolites (*n* = 1,157) and lipids (*n* = 514) were obtained after data curation and normalization.

**Fig. 2:**
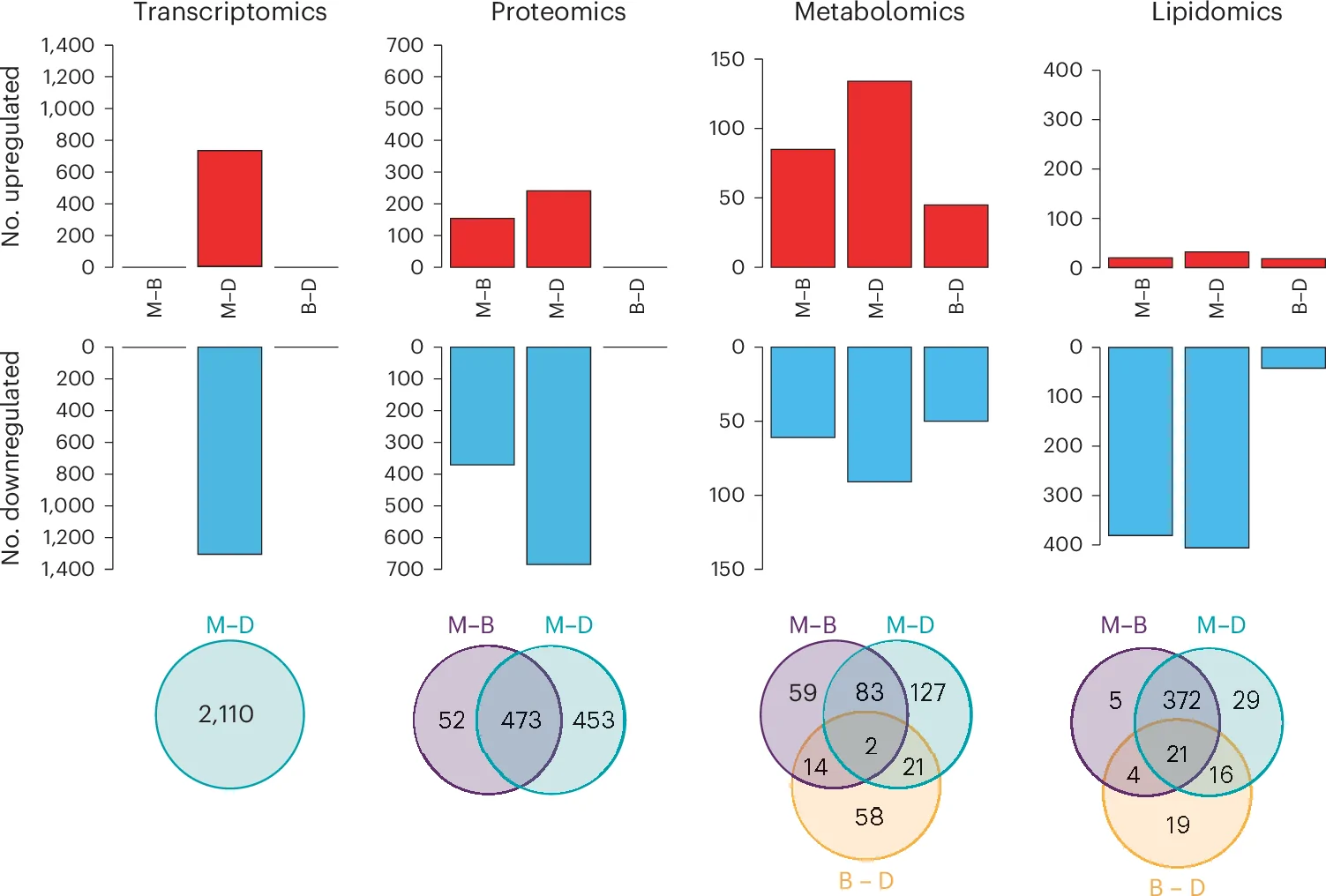
Differentially abundant molecules per -omic. Top, histograms of the number of differentially abundant molecules per -omic analysis and contrast (M–B, M–D and B–D) separated by upregulated (red) and downregulated (blue) directionality (for example, red or upregulated in M–D indicates increasing abundance from mucosa to dysplastic polyp). Differential abundance was defined by an FC of ±15% and FDR of 5% for the transcriptome, an FC of ±15% and FDR of 1% for the proteome and a *P* value of 5% for the lipidome and metabolome. Bottom, Venn diagrams show the degree of differentially abundant molecules shared between the contrasts regardless of directionality for each single -omic. The absence of a bar (histogram) or circle (Venn diagram) means that no significant analytes were identified. The statistical tests used were two-sided Wald tests for the transcriptomic, lipidomic and metabolomic data and one-sided *F*-tests for the proteomic data. Source data

**Extended Data Fig. 1:**
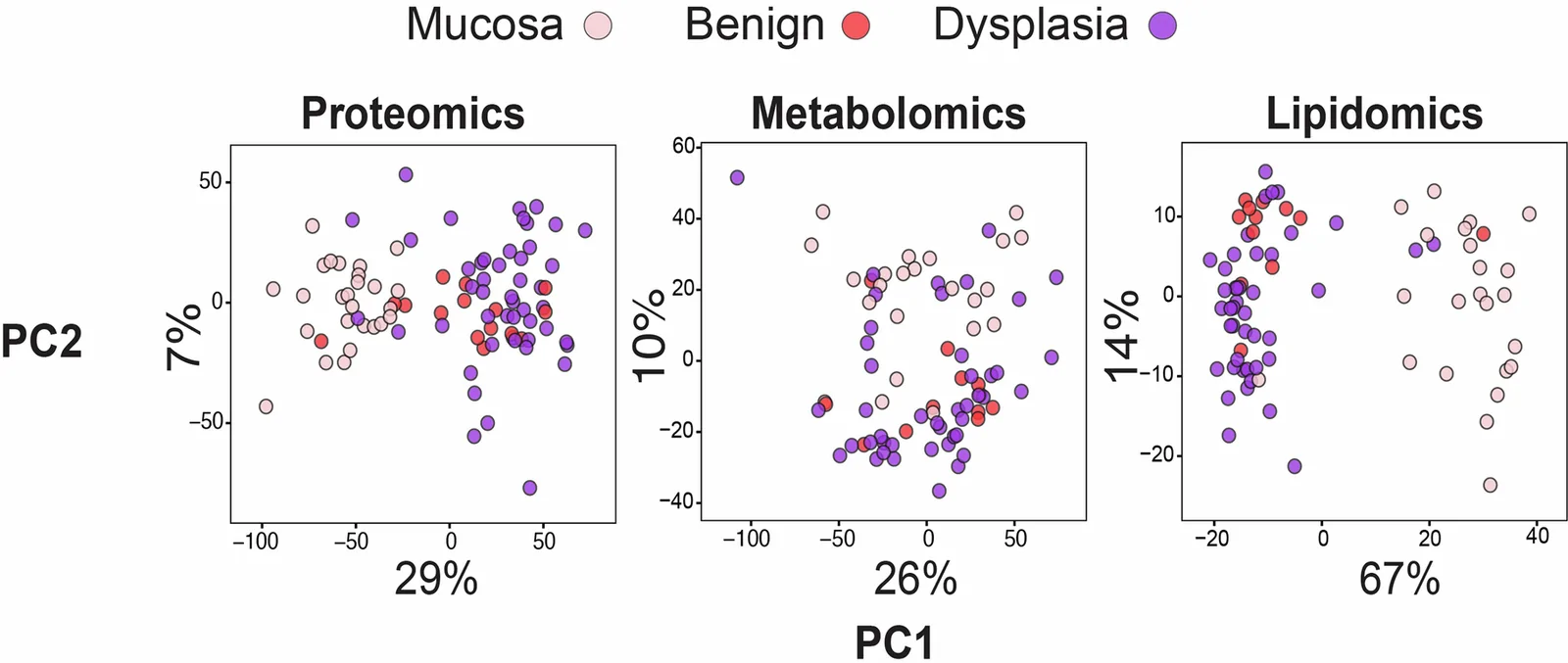
Omic PCA Plots. Principal component analysis across -omic assays (transcriptome is omitted due to technical effects).

**Extended Data Fig. 2:**
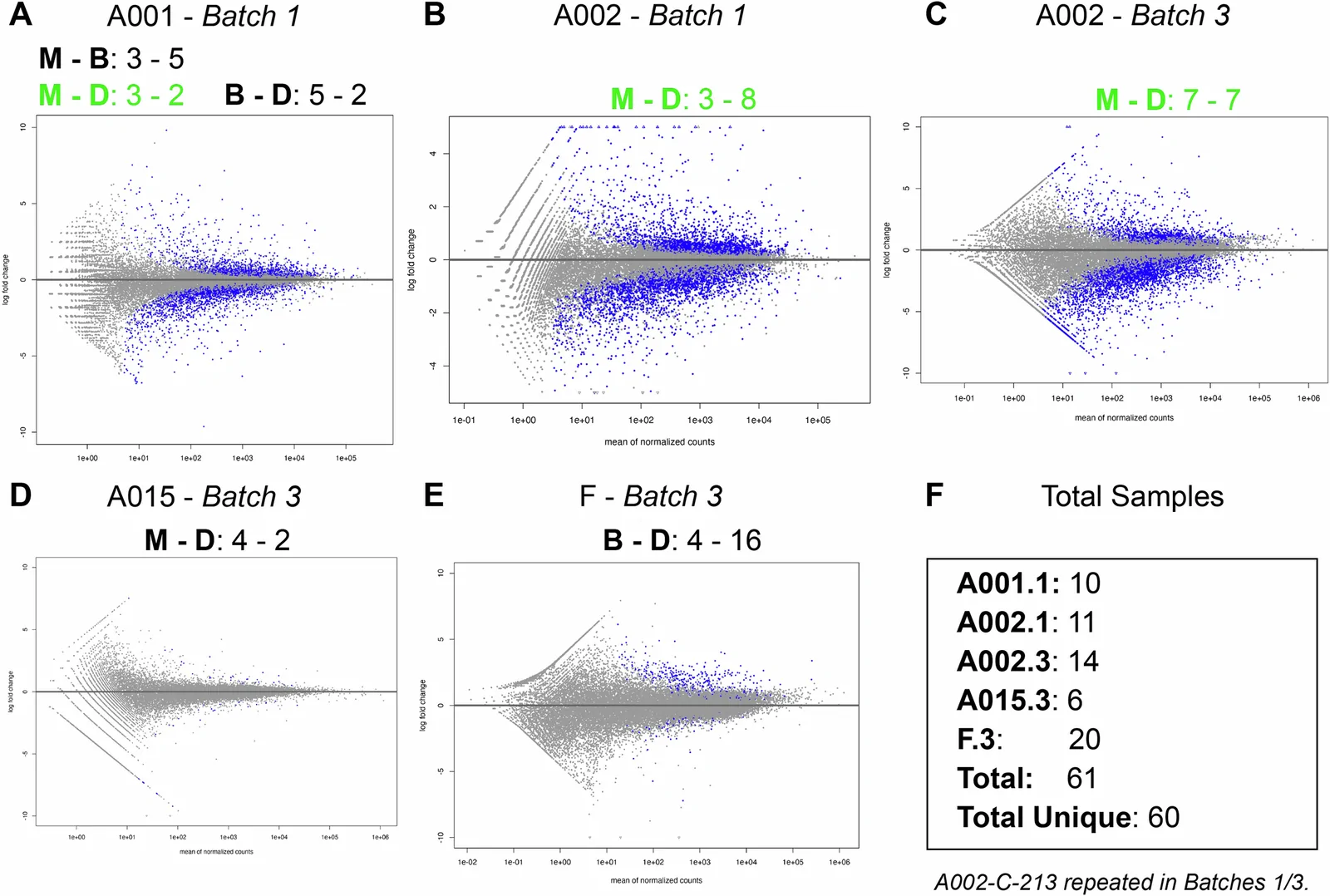
DESeq2 Plots. Transcriptomic DESeq2 MA plots from various (Patient, Batch) pairs along with the number of samples from which mRNA was derived for each of the three stages and contrasts. Green indicates contrasts that ultimately contribute to differential expression. (**a**) MA plot for patient A001 in Batch 1 with 10 samples (3 Mucosa, 5 Benign Polyp, 2 Dysplastic Polyp). (**b**) MA plot for patient A002 in Batch 1 with 11 samples (3 Mucosa and 8 Dysplastic Polyp). (**c**) MA plot for patient A002 in Batch 3 with 14 samples (7 Mucosa and 7 Dysplastic). (**d**) MA plot for patient A015 in Batch 3 (4 Mucosa and 2 Dysplastic Polyps). (**e**) MA plot for patient F in Batch 3 (4 Benign Polyp and 16 Dysplastic Polyp). (**f**) Shows the total breakdown in the number of samples from which mRNA was extracted across the different (Patient, Batch) pairs. Though there were 61 samples, one sample (A002-C-213) was present in two batches.

Principal component analysis (PCA) of each -ome revealed a delineation among mucosa, benign polyps and dysplastic polyps, suggesting a progression of malignancy in precancer colonic tissue (transcriptomic PCA was omitted because of technical reasons; Methods); little difference was observed between benign and dysplastic polyps, while mucosa samples were clearly separated from both categories of polyp (Extended Data Fig. 1). Differential analysis was performed for each -ome between each pair of transitions between the three precancerous stages: mucosa versus benign polyp (M–B), mucosa versus dysplastic polyp (M–D) and benign polyp versus dysplastic polyp (B–D). Differential analysis revealed thousands of molecular changes (Fig. 2 and Supplementary Table 1a–d; *P* < 0.05). The largest numbers of molecular changes were found for transcripts (*n* = 2,110, 8.97% of 23,512 tests for one transition) and proteins (*n* = 1,451, 3.9% of 37,167 tests for three transitions) and the fewest were found for metabolites and lipid components (*n* = 486, 14% of 3,471 tests and *n* = 900, 52.5% of 1,713 tests across three transitions, respectively; Fig. 2 and Supplementary Table 1a–d). The number of unique differential molecules by -ome was as follows: transcriptome (*n* = 2,110, 8.97% of 23,512 transcripts), proteome (*n* = 978, 7.9% of 12,389 proteins), metabolome (*n* = 364, 31.5% of 1,157 metabolites) and lipidome (*n* = 466, 90.7% of 514 lipids). More downregulated molecules were identified than upregulated molecules (that is, less abundant in the more advanced cancerous stage). At the gene expression level (Supplementary Table 1a), most changes were evident in the M–D transition, as technical issues did not permit the identification of changes in the other transitions with statistical reliability (Extended Data Fig. 2). Similarly, the most significant differences in the proteome, lipidome and metabolome (Supplementary Table 1b–d) occurred between mucosa and polyp, whether M–B or M–D. Differential analysis was performed across all participants, but participant-specific differential analysis was also performed (Methods; the experimental design and statistics of differential analysis are shown in Supplementary Table 4a–g and the differential transcripts, proteins, metabolites and lipids are reported in Supplementary Table 5a–d); the differential analysis henceforth refers to the differential analysis across all participants.

**Fig. 3:**
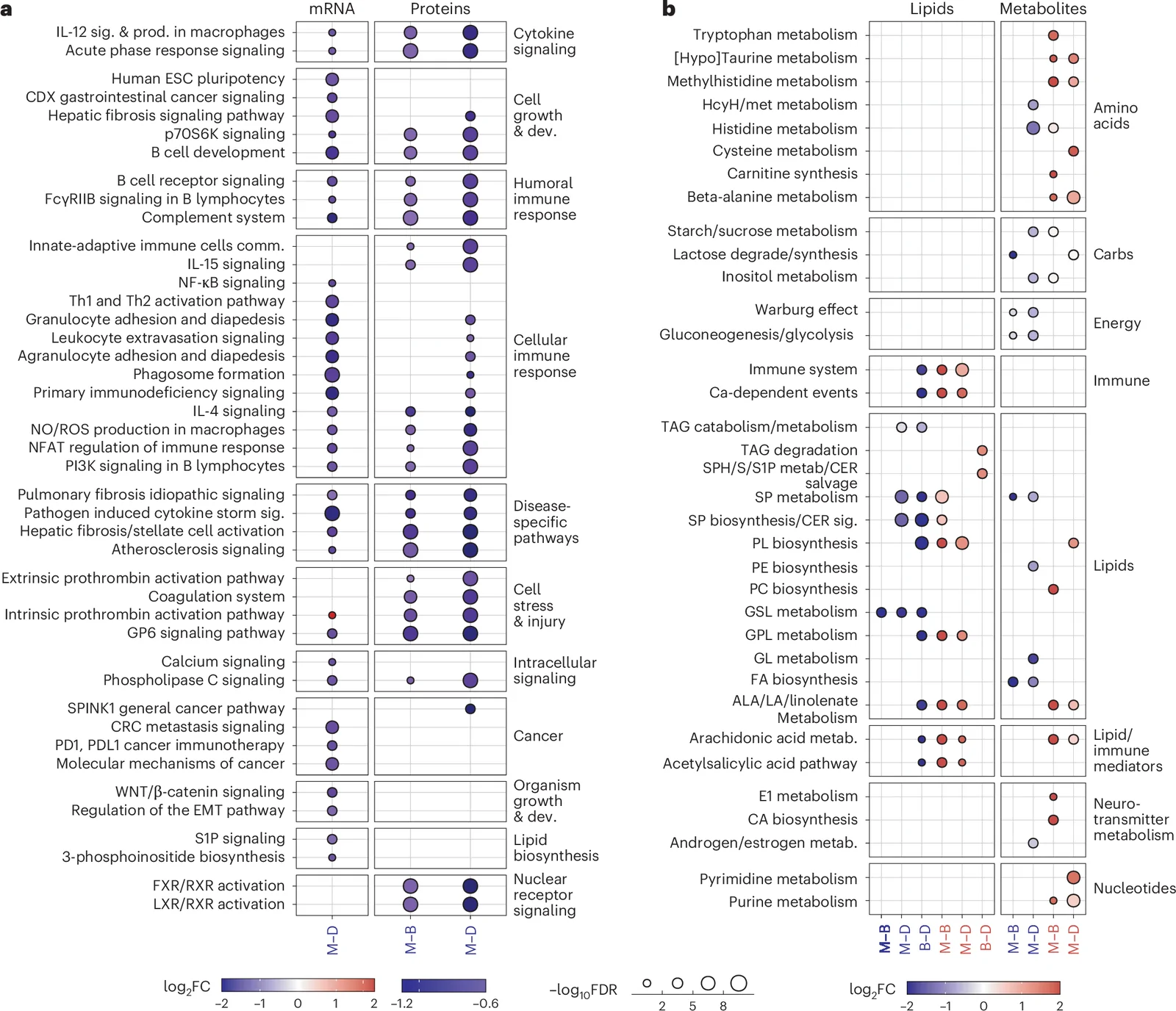
Selected pathway enrichments run for differentially abundant molecules for each -omic. Each dot indicates an enrichment of a pathway for a particular -omic; its size indicates the −log_10_FDR of the enrichment (a larger dot indicates a smaller adjusted *P* value) and the color indicates the median log_2_FC. **a**, Enrichment analyses for the transcriptome and proteome (separately) were performed using IPA and upregulated and downregulated molecules were enriched separately, although only downregulated enrichments were significant. Pathways were selected that reflected strong agreement between the proteome and transcriptome and were distributed across a variety of cellular and molecular functions relevant to CRC. **b**, Enrichment analysis for the lipidome and metabolome used the CPDB and MSEA tools rather than IPA and, similarly to the transcriptome and proteome, selected pathways spanning a variety of molecular and cellular functions relevant to CRC were selected (for example, arachidonic acid). Enrichments were performed with all differential molecules and pathways were deemed upregulated and downregulated according to the median log_2_FC of the enriched molecules. E1, estrone; CA, caffeine; ALA, alpha-linolenic acid; LA, linoleic acid; PL, phospholipid; S, sphingosine; HcyH, homocysteine; Met, methionine; [Hypo]Taurine, taurine and hypotaurine. Source data

**Fig. 4:**
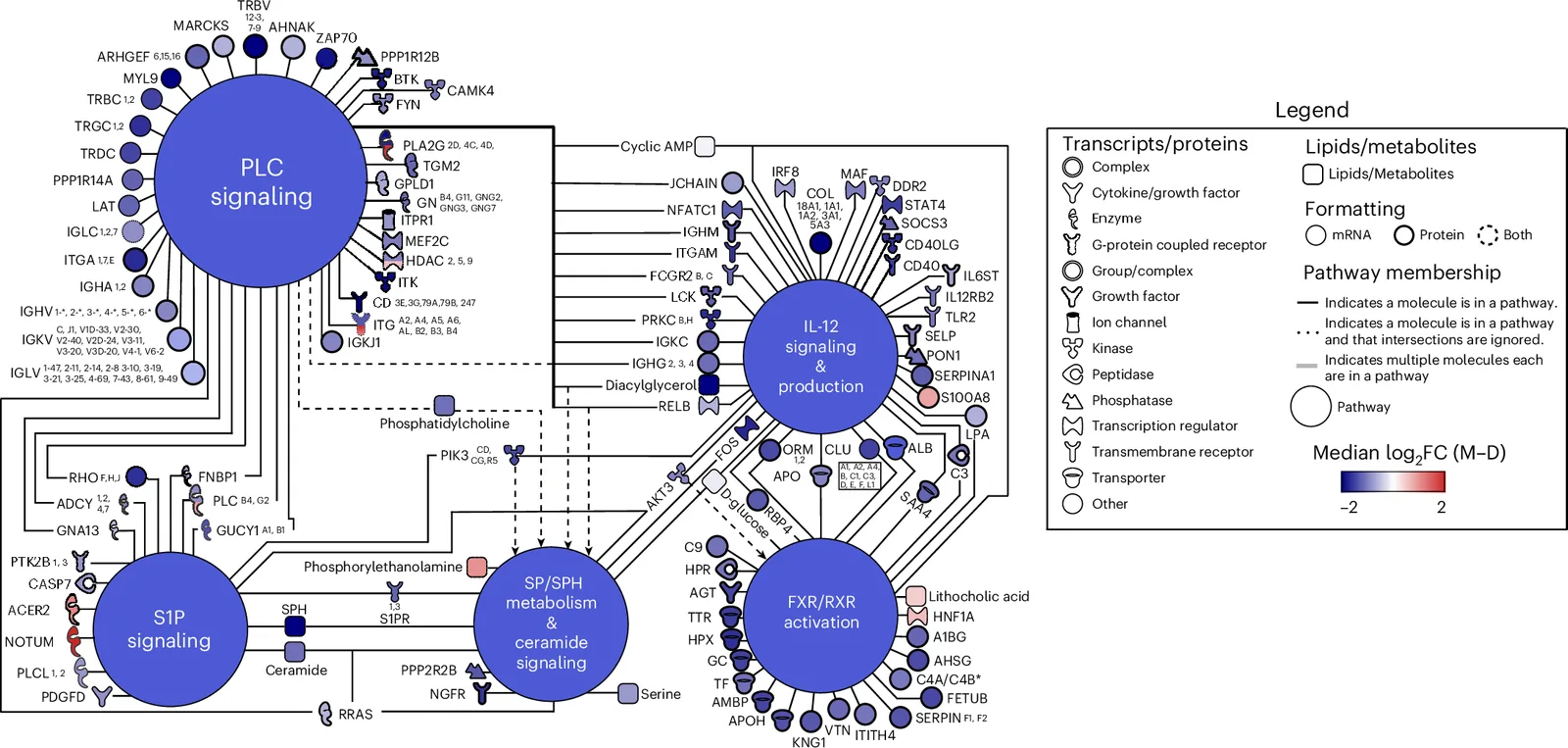
A multiomic diagram describing the relationship between various enriched pathways in the M–D transition in terms of the hundreds of molecules they share in common. The molecules are colored according to the log_2_FC and are generally shaped according to their molecular function. Lipids and metabolites are both symbolized as chemicals, while the remaining molecules are genes or proteins. Some molecules have both gene and protein information and are outlined with dots; otherwise, a bold outline indicates that the molecule is a protein. To reduce the complexity, molecules with similar prefixes and belonging to the same family were combined; subscript characters denote the different members of the family and the molecule complex is colored by the median log_2_FC. In situations where a complex contains both upregulated and downregulated members, half the molecule is colored with the median log_2_FC for one direction and the other half of the molecule is colored with the median log_2_FC for the other direction. Large circles are pathways from IPA, MSEA or CPDB ORA and are colored blue to indicate that all are downregulated from M–D. Lines flow from molecules to pathways to indicate membership. Thick lines that intersect with multiple thin lines simplify the flow of information and indicate that all thin lines move along the direction of thick lines. Lines that are dotted obviate lines they so happen to intersect. The asterisk collapses the following molecules with the IGHV (immunoglobulin heavy chain variable region) prefix: 1-18, 1-2, 1-46, 1-69, 2-26, 2-5, 2-70D, 3-13, 3-15, 3-30, 3-35, 3-43, 3-49, 3-7, 3-74, 4-28, 4-34, 4-4, 5-51 and 6-1. Source data

Differentially abundant transcripts and proteins across the three precancerous transitions were analyzed using the Qiagen Ingenuity Pathway Analysis (IPA). Enrichments were performed separately for upregulated and downregulated molecules. Similar analyses were performed for lipid and metabolite species using ConsensusPathDB (CPDB)^11^ overrepresentation analysis (ORA^12^) and metabolite set enrichment analysis (MSEA^13^). Across all -omes and transitions, a total of 824 pathway enrichments were detected at a false discovery rate (FDR) of 5% (Supplementary Table 2), with 431 unique enriched pathways (Figs. 3 and 4a). These pathways cover a diverse set of biological areas (for example, immune, cancer and cell signaling). Because the number of pathways is extremely large only several of the key pathways are highlighted in later sections.

### M–B comparison

Comparing benign polyps to FAP mucosa identified 1,347 altered molecules (402 lipids, 420 metabolites, 525 proteins and 0 transcripts; Extended Data Fig. 2 and Methods) and over 100 altered pathways (175 pathway enrichments consisting of 162 unique pathways at FDR ≤ 5%; Supplementary Table 2). In the proteome (Fig. 3a), all M–B significant pathways were enriched for downregulated proteins; the figure shows 24 of 39 enriched pathways. The most enriched pathway (−log_10_FDR = 21.70) was acute-phase response signaling, a cytokine signaling-related pathway. It was one of many immune-related (cellular and humoral immune response and cytokine signaling) pathways (*n* = 15) in the 39. Only one pathway of the 39, glutathione redox reactions I (−log_10_FDR = 2.04) involved in biosynthesis, was unique in the M–B transition (Supplementary Table 2). Other top proteomic pathways (Fig. 3a) included nuclear receptor signaling pathways LXR and RXR activation (−log_10_FDR = 20.40) and FXR and RXR activation (−log_10_FDR = 17.90), a cellular stress or injury GP6 signaling pathway (−log_10_FDR = 12.50), a humoral immune response complement system (−log_10_FDR = 11.80) pathway and disease-specific pathways atherosclerosis signaling (−log_10_FDR = 11.20) and hepatic fibrosis and hepatic stellate cell activation (−log_10_FDR = 10.70). Key pathways related to CRC growth and treatment included phospholipase C (PLC) signaling^14^ (represses CRC growth) and interleukin (IL)-12 signaling and production in macrophages^15^ (increases radiosensitivity in mouse models of colon cancer because of increases in IL-12-dependent T helper cell 1 response).

Metabolites and lipids were also significantly dysregulated. Amino acids, nucleotides and neurotransmitters, notably, *S*-adenosyl-homocysteine, adenine, deoxyadenosine, xanthosine, nicotinamide adenine dinucleotide and citicoline were generally elevated, although histamine, a histidine product, decreased in this transition. Fatty acids (FAs), lipids and lipid immune mediators were also generally elevated, including docosahexaenoic acid, adrenic acid, linoleic acid, eicosatrienoic acid, hydroxyoctadecadienoic acid, eicosadienoic acid, docosapentaenoic acid, 5-hydroxyeicosatetraenoic acid, 1-(*sn*-glycero-3-phospho)-1d-*myo*-inositol, cyclic phosphatidic acids, glycolipids (phosphatidylcholine (PC), lysophosphatidylcholine (LPC), phosphatidylethanolamine (PE) and lysophosphatidylethanolamine (LPE)) and cholesterol esters (CEs). Downregulated molecules notably consisted of ceramides (CERs), sphingolipids (SPs), diglycerols and triglycerols, although exceptions were evident such as elevation of LacCer(d18:1/18:0). The known role of these molecules suggests potential implications for the regulation of inflammatory and immune pathways.

There were 35 pathways or pathway groups from 31 and 105 metabolomic and lipidomic enriched pathways in the M–B transition (Fig. 3b). SP metabolism (−log_10_FDR = 5.2) and arachidonic acid metabolism (metabolome, −log_10_FDR = 2.96; lipidome, −log_10_FDR = 2.0) were the most enriched lipid and metabolic pathways, respectively (five and six pathways had equivalent −log_10_FDR, respectively). Dysregulation of the arachidonic acid pathway is particularly interesting because it is involved in inflammation and can be suppressed by aspirin or other nonsteroidal anti-inflammatory drugs (NSAIDs), a treatment for persons with FAP to slow polyp progression and reduce CRC formation^16–18^. The pathway was upregulated in both lipidome and metabolome data for the M–B transition. Furthermore, the acetylsalicylic acid (ASA) pathway, which interacts with arachidonic acid, was also enriched for upregulated lipids in our dataset. Thus, our results provide a molecular explanation for the therapeutic strategy for FAP treatment. Altered pathways related to CER and SP metabolism and signaling were also altered in the lipidome; SP changes were previously shown to be evident in colon cancer^19^. CER and sphingosine are two important lipid molecules that have notable effects on T cell function^20^.

Strong lipidomic changes were observed in the pathway titled ‘immune system’ (Reactome; −log_10_FDR = 5.12, driven by CERs, CEs and glycerophospholipids (GPLs) (Supplementary Table 1c)). We also observed lipidomic alterations in Ca-dependent events (Reactome; −log_10_FDR = 3.30, driven by increases in PC and LPC (Supplementary Table 1c)), with Ca as a vital component of the immune system and intimately involved in the arachidonic acid pathway. PCs and LPCs are major components of cell membranes and also serve as reservoirs of FAs for energy and signaling^21^. Glycerolipids (GLs) can affect the localization or activity of Ca-binding proteins, Ca channels or other components of the Ca signaling pathway^22^ and, thus, impact processes such as immune cell migration, cytokine release and other immune functions. Lastly, despite a lack of Kyoto Encyclopedia of Genes and Genomes (KEGG)-identified pathway enrichments related to triacylglycerol (TAG) biology emerging in this analysis, the extensive alteration of TAG (Supplementary Table 1c) species in the M–B transition is noteworthy and relevant when comparing to later transitions.

### M–D comparison

Large numbers of molecular (3,707 sourced from 2,110 transcripts; Extended Data Fig. 2 and Methods; 926 proteins, 438 lipids and 233 metabolites) and pathway (485 from 395 unique at FDR ≤ 5%; Supplementary Table 2) changes were also observed during the comparison of dysplastic polyp to mucosa. This transition accounted for the highest number of molecular and pathway changes (Fig. 2) across the three comparisons and was the only comparison that identified differential transcripts detected across more than a single batch or participant (Extended Data Fig. 2). Differentially expressed molecules were often shared between the M–B and M–D transitions for the lipidome, metabolome and proteome (Fig. 2). Key pathways are shown in Fig. 3.

### Proteomic and transcriptomic pathway enrichments

Consistent with the M–B results, nearly all transcriptomic and proteomic pathways for M–D were downregulated (except the transcriptomic M–D enriched pathway intrinsic prothrombin activation, which was upregulated with −log_10_FDR = 1.30, marginally meeting the 5% FDR cutoff). Overall, 70 pathways of a total of 212 transcriptomic and 93 proteomic pathways were enriched across both modalities (Supplementary Table 2). Of the 98 total proteomic pathways, 38 were also seen in the M–B proteomic comparison.

The five most enriched transcriptomic pathways were the disease pathway pathogen-induced cytokine storm signaling (−log_10_FDR = 10.20), the cellular immune response pathway phagosome formation (−log_10_FDR = 9.54) and the organismal growth and development pathways axonal guidance signaling (−log_10_FDR = 9.47), cardiac hypertrophy signaling (enhanced) (−log_10_FDR = 9.47) and sperm motility (−log_10_FDR = 9.25); all pathways but axonal guidance signaling were supported by the proteome. The five most enriched proteomic pathways were cytokine signaling pathway acute-phase response signaling (−log_10_FDR = 33.30), cell growth and proliferation pathway B cell development (−log_10_FDR = 32.30), nuclear receptor signaling pathway LXR and RXR activation (−log_10_FDR = 27.70) and humoral immune response pathways complement system (−log_10_FDR = 26.80) and FcγRIIB signaling in B lymphocytes (−log_10_FDR = 24.70); all pathways but acute-phase response signaling were supported by the transcriptome for M–D (Supplementary Table 2). Notably, 57 and 29 M–D transcriptomic and proteomic pathways, respectively, were involved in the immune system (cytokine signaling, cellular immune response or humoral immune response), with 23 overlapping between the two. The 39 pathways depicted in Fig. 3a display a selection of enriched pathways with high concordance across transitions in the proteome and between the transcriptome and proteome; these pathways were among the most significant and relevant enrichments spread across a diverse group of cellular and molecular functions, including pathways relevant to CRC such as CRC metastasis signaling (−log_10_FDR = 6.03), PLC signaling and FXR and RXR activation, which was shown to antagonize Wnt and β-catenin signaling in CRC tumorigenesis^23^. Thus, the transcriptome and proteome showed a high degree of concordance in enrichments in the M–D transition and even between the M–B and M–D transitions in the proteome and all such enrichments (except for one) consisted of downregulated genes or proteins; these enrichments spanned a variety of cellular and molecular functions and many were related to CRC.

### Lipidomic and metabolomic pathway enrichments

The M–D transition in persons with FAP highlighted extensive changes within both the metabolome and the lipidome profiles (Fig. 2); many of these were similar to those observed during the M–B transition but their magnitude in numbers and levels was usually greater. We observed an increase in both nucleotides and their derivatives, such as cytidine, cytosine, cytidine diphosphate and deoxyuridine, alongside amino acids and their derivatives. Notably, higher levels of specific amino acids and their derivatives such as serine, asparagine and deaminohistidine (imidazolepropionic acid) were evident. The M–D transition also exhibited an extensive number and increased level of CEs and GLs, accompanied by a higher number of decreased SPs, CER, monoglycerol, diglycerol and triglycerol. Interestingly, leukotriene A4, which was found to be increased during the M–D transition, along with 5-hydroxyeicosatetraenoic acid, has an important role in the arachidonic acid pathway, contributing to immune cell function.

Examination of the lipidomic and metabolomic pathway enrichments in Fig. 3b and Supplementary Table 2 reveals enrichments of both upregulated and downregulated metabolite and lipid sets for M–D. Between the 144 lipidome and 37 metabolome enrichments at FDR ≤ 5%, only three metabolomic pathways were enriched for the nearest analog in the lipidome in the M–D transition: arachidonic acid (metabolome, −log_10_FDR = 3.40; lipidome, −log_10_FDR = 1.94), phospholipid biosynthesis (metabolome, −log_10_FDR = 3.40; lipidome, −log_10_FDR = 5.81) and SP metabolism (metabolome, −log_10_FDR = 4.81; lipidome, −log_10_FDR = 7.24). In the metabolome, the six most enriched pathways were primarily related to amino acid and nucleotide metabolism: histidine metabolism (−log_10_FDR = 6.82), β-alanine metabolism (−log_10_FDR = 6.57), pyrimidine metabolism (−log_10_FDR = 6.54), pterine biosynthesis (−log_10_FDR = 6.30), arginine and proline metabolism (−log_10_FDR = 5.66) and purine metabolism (−log_10_FDR = 5.49). The six most enriched lipidomic pathways were naturally related to lipid metabolism and biosynthesis: methylenetetrahydrofolate reductase (MTHFR) deficiency (−log_10_FDR = 7.53), SP metabolism (−log_10_FDR = 7.24), SP de novo biosynthesis (−log_10_FDR = 6.90), high-density lipoprotein remodeling (−log_10_FDR = 6.09), plasma lipoprotein assembly, remodeling and clearance (−log_10_FDR = 6.09) and phospholipid biosynthesis (−log_10_FDR = 5.81) (Supplementary Table 2).

These altered metabolomic and lipidomic pathways are expected to have an abundance of molecular and cellular consequences. Metabolic decreases in the histidine pathway are reported to induce a decrease in amino acid oxidation and a decrease in protein turnover^24^; altered histidine metabolism also attenuates antioxidant defense, redox balance and signaling^25,26^. Nucleotide metabolic pathways are crucial for cell division^27^ and are shifted the M–D transition, presumably to support rapid cell rapid cell proliferation^28^. Alteration of SP metabolism is observed in the M–D transition and has connections to immune cell function^20^.

Similarly to the M–B transition, dysregulation of lipid metabolism and immune cell response emerged in the M–D transition as the most prominently altered pathways enriched in both the metabolome and the lipidome of persons with FAP. Enrichment of arachidonic acid and linoleic acid metabolism, both of which intersect with immune regulation^29,30^ (Fig. 3b), was observed for the M–B transition. Matching against the Reactome database revealed an increased number of GLs and enrichment of diacylglycerol (DAG) and Ca^+^-dependent events in the M–D transition (Fig. 3b and Supplementary Table 2). A higher proportion of TAG species was also observed in the M–D transition compared to the M–B transition. This shift in the abundance of TAG species could indicate a notable metabolic shift that is likely related to changes in energy use, signaling pathways or other cellular processes.

Figure 3b also showcases other important lipidomic and metabolomic pathways that were enriched in M–D and spanned a broad number of molecular and cellular functions. As found for the M–B transition, notable downregulated enriched lipid pathways and pathway groups for M–D were SP metabolism (Wikipathways, −log_10_FDR = 7.24), SP biosynthesis (Reactome, −log_10_FDR = 6.90), CER signaling (BioCarta, −log_10_FDR = 4.61) and glycosphingolipid (GSL) metabolism (Reactome, −log_10_FDR = 3.67), which was also downregulated enriched for M–B. Among upregulated enriched lipid pathways and pathway groups for M–D were immune-related pathways, Ca-dependent events and immune system, lipids acting as immune mediators, arachidonic acid and ASA (aspirin) pathways and lipid-related pathway groups (GPL metabolism, phospholipid biosynthesis and alpha-linolenic acid, linoleic acid and linoleate metabolism). Some of these pathways are related to CRC. For instance, Ca-dependent events such as Ca^2+^ remodeling is associated with CRC. Ca^2+^ also interacts with aspirin, which inhibits CRC by blocking CRC from free intracellular Ca^2+^ (ref. ^31^). Ca is also relevant to PLC because it is ejected from intracellular compartments in T cells during PLC activation by DAG, protein kinase C (PKC) and inositol 1,4,5-trisphosphate^32^. PLC is important for regulating both Ca and phosphoinositide^33^. Ca-dependent events are connected intimately with other molecules and pathways associated with CRC. In addition, SP biosynthesis is related to CRC; an increase in the ratio of sphingosine-1-phosphate (S1P) to CER is associated with cancer progression^19^. We found S1P signaling in the transcriptome and CER signaling in the lipidome to be enriched in the M–D transition and, interestingly, both S1P and CER are critical components for the formation of arachidonic acid^34^. The high connectivity of the molecules in the S1P and CER signaling and PLC signaling pathways are shown in Fig. 4 for the M–D transition. Notable downregulated enriched metabolite pathways and pathway groups for M–D included FA biosynthesis, SP metabolism, inositol metabolism and gluconeogenesis; notable downregulated enriched metabolite pathways and pathway groups for M–D included purine and pyrimidine metabolism, arachidonic acid, alpha-linolenic acid, linoleic acid and linoleate metabolism and assorted amino acid pathways. These pathways are relevant to CRC in a fashion similar to that described for the lipidome.

### Multiomic pathways and networks characterizing M–D transition

To illustrate the connections between CRC related pathways in the M–D transition, six such pathways (IL-12 signaling and production in macrophages, PLC signaling, FXR and RXR activation, SP and sphingomyelin (SPH) metabolism, CER signaling and S1P signaling) were selected and a multiomic pathway diagram was constructed (Fig. 4). Numerous proteins and transcripts relevant to CRC emerged as differentially altered including downregulated Serpin family members such as *SERPINF2* (ref. ^35^) and *SERPINA1* (refs. ^35,36^) (although these molecules are upregulated in adenomas and cancer relative to mucosa), upregulated *NOTUM* (ref. ^37^) and upregulated *S100A8* (refs. ^38,39^). Several lipids and metabolites whose downregulation is associated with CRC tie SP membrane metabolism and signaling pathways together explicitly, such as CER and SPH.

CER is a proapoptotic anticancer SP synthesized in several pathways, one of which is an alternative salvage pathway that converts SPH to CER^40^; the biosynthesis of SPs and signaling of CER were both enriched in the M–D transition but CER itself was downregulated. Additionally, even though the CER salvage pathway was not enriched until the B–D transition, SPH was downregulated in the M–D transition, indicating that there may be evidence of compensatory lipidomic and metabolomic mechanisms to halt the progression of CRC in precancerous tissue. Not only do lipids and metabolites connect these pathways but two important receptors detected in the transcriptome also do; S1P receptors S1PR1 and S1PR3 connect S1P signaling with SP and SPH metabolism and CER signaling and were downregulated in the M–D transition. Classically, activation of S1P signaling heralds the progression of cancer in colonic tissue^41^. The same is generally true of the receptors S1PR1 and S1PR3; however, the literature is not in complete agreement on this^41^ and it may be that the precancerous dysplastic polyps are not advanced enough to evince upregulation of these receptors at the mRNA level. Other important molecules connecting CRC-associated pathways include *PIK3CD*, which normally upregulates protein kinase B, glycogen synthase kinase 3β and β‐catenin and, thus, colon cancer growth and proliferation^42^. However, as shown in Fig. 4, in the M–D transition, both *AKT3* and *PIK3CD*, which connect IL-12 signaling with SP and SPH metabolism and CER signaling, were downregulated, again re-emphasizing that dysplastic tissue does not yet exhibit the full characteristics of CRC. Overall, Fig. 4 shows the numerous connections of key molecules governing important pathways that occur early in cancer formation and that the molecules between these pathways were overwhelmingly downregulated.

**Fig. 5:**
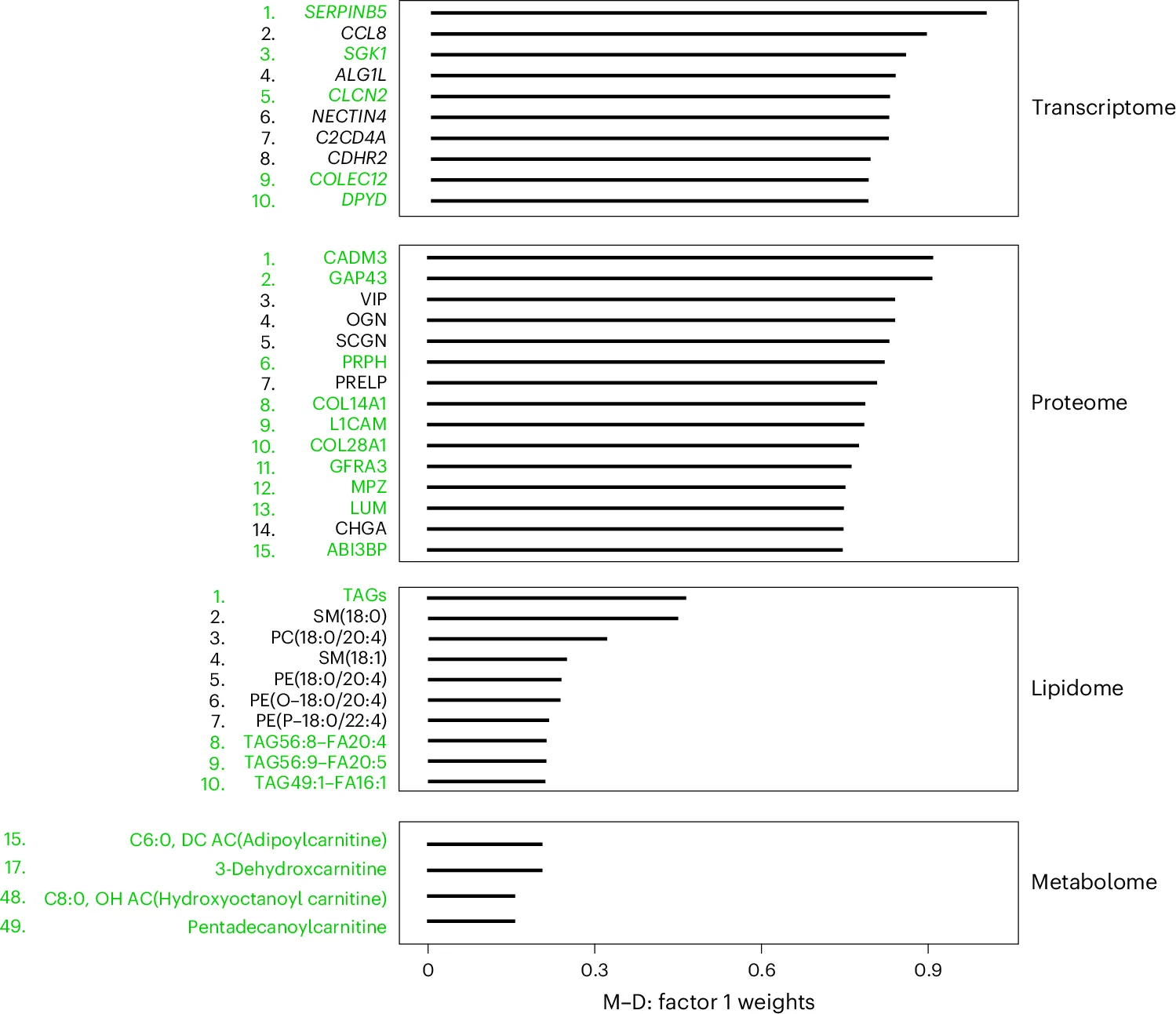
Selected molecules from MOFA with high weight alongside the latent factor (factor 1) correlated with dysplasia. Relevant molecules are highlighted in green. The top ten and four of the top 50 molecules for the lipidome and metabolome are shown, respectively. The four metabolites are all carnitines. Source data

**Extended Data Fig. 3:**
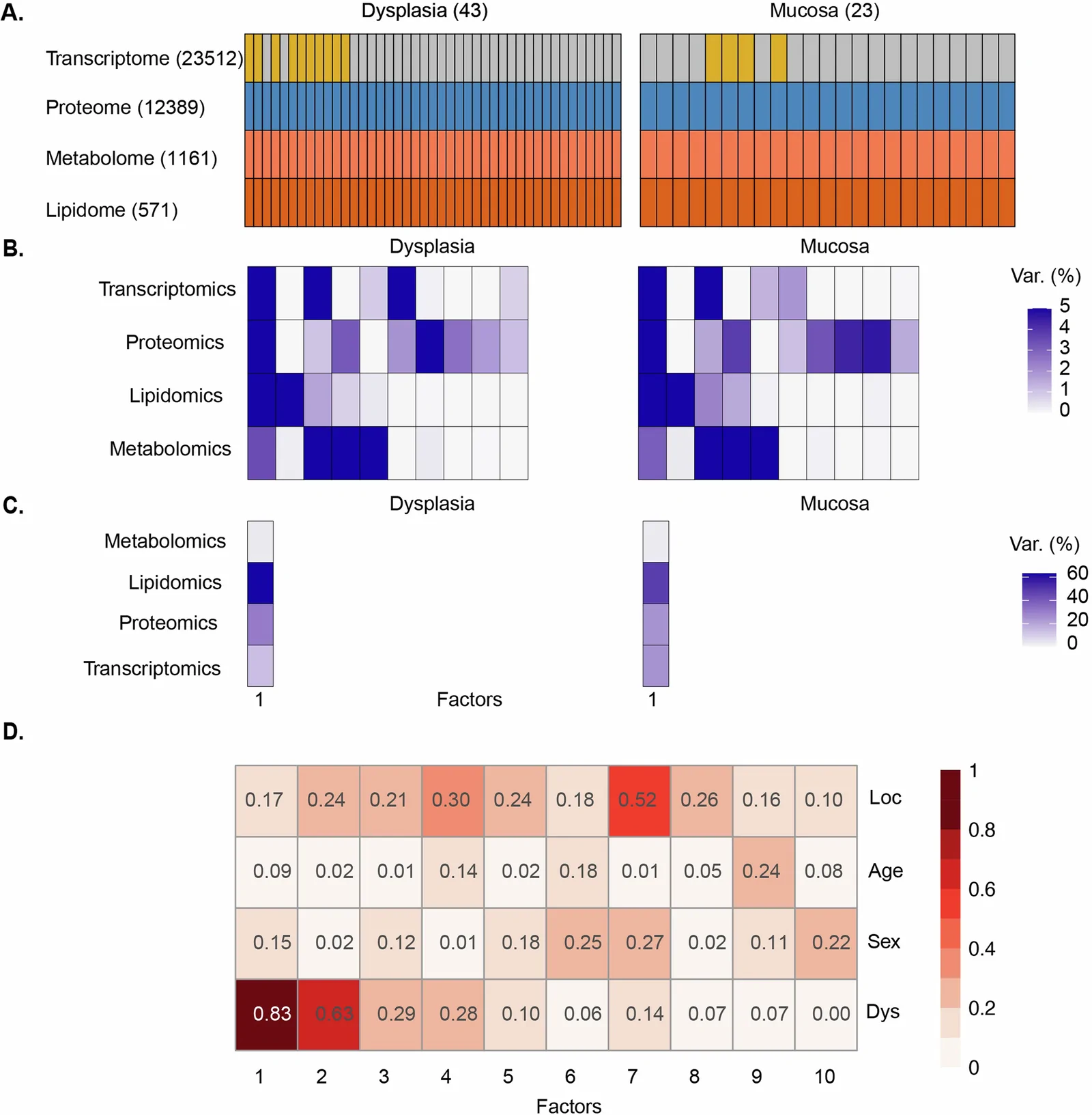
MOFA Statistics. Multi-omic Factor Analysis (MOFA) statistics for 10 factors. (**a**) Shown are the 4 -omic modalities and their number of molecules. On the left are 43 Dysplastic Polyps shown (10 for transcriptome) and on the right are 23 Mucosa samples (4 for the transcriptome). (**b**) Shows the % variance explained by each -omic and factor for the Dysplastic and Mucosa Samples. (**c**) Show the % variance explained by the 1st factor for the Dysplastic and Mucosa samples. (**d**) Shows the correlation coefficient of each factor with several metadata variables including dysplasia (Dys). Factor 1 is highly correlated with Dysplastic status and molecules separated at the extremes of this axis are implicated with dysplasia and investigated.

**Extended Data Fig. 4:**
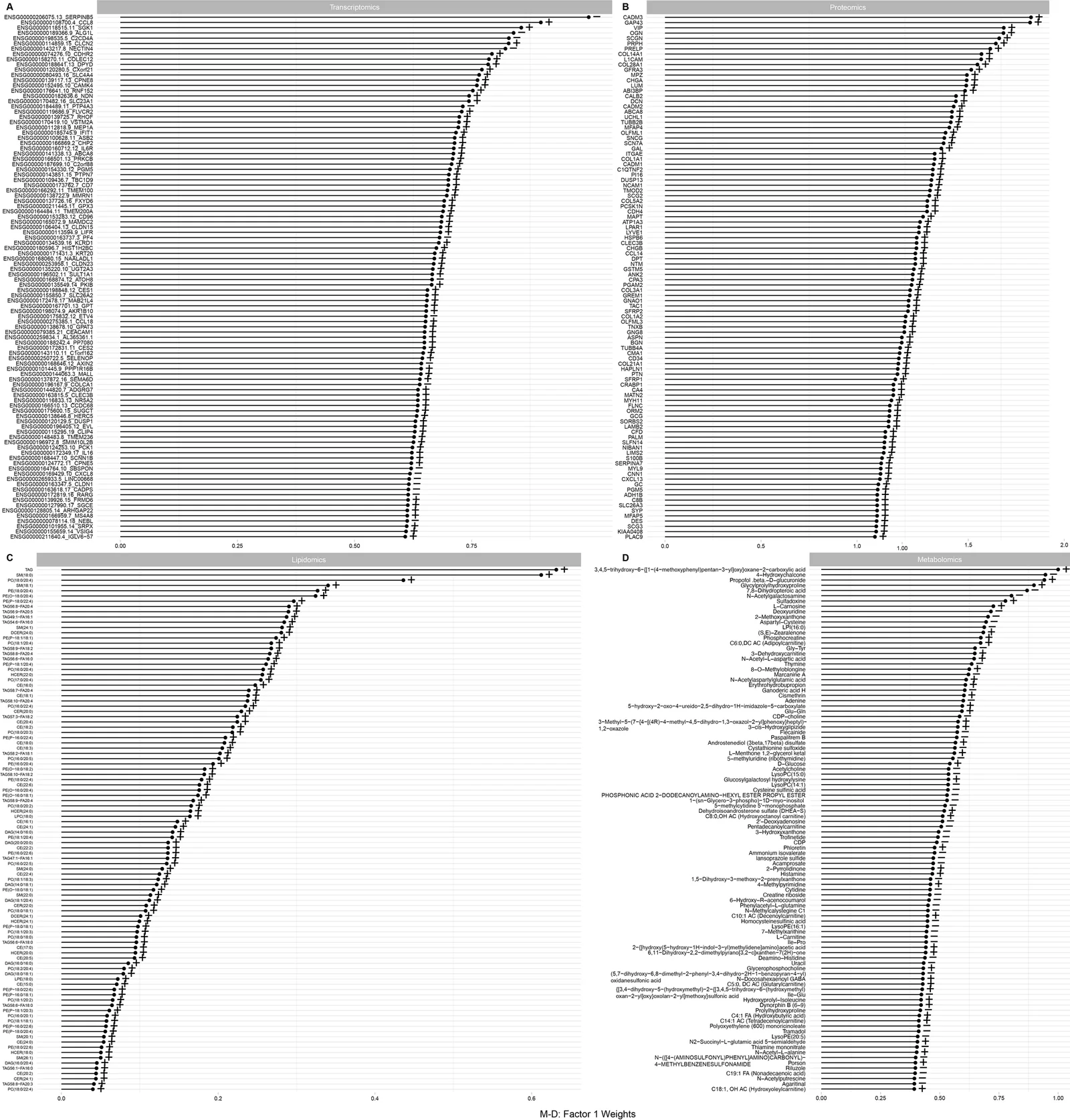
MOFA Factor 1 Omic Rankings. The top 50 transcripts (**a**), proteins (**b**), lipids (**c**), and metabolites (**d**) along dysplasia correlated factor 1 in MOFA.

The multiomic data were further subject to analysis by inferring a latent axis correlated with dysplastic progression (Extended Data Figs. 3 and 4) along which molecules of all molecular types covaried (Fig. 5). This method identified the top covarying transcripts, proteins, lipids and metabolites associated with dysplasia (Fig. 5). Verification was restricted to the top ten transcripts along the latent factor and half had some connection to CRC. *SERPINB5* is involved in CRC progression; it depends on and covaries positively with the cancer marker carcinoembryonic antigen^43^. *SGK* is involved in cell survival, proliferation and ion transport and has been implicated in CRC progression^44,45^. *CLCN2* expression is reduced in CRC compared to normal colonic tissue^46^. *NECTIN4* is a cell adhesion molecule involved in cell–cell interaction that is dysregulated in CRC^47^ and *COLEC12* attenuates stem cell-like features in CRC on knockdown^48^. Lastly, *DPYD* is related to CRC treatment response^46^.

Of the top 15 covarying proteins, those encoded by *CADM3*, *GAP43*, *PRPH*, *L1CAM*, *GFRA3*, *MPZ* and *AB13BP* are all neural related, indicating other roles in precancer, while those encoded by *COL14A1* and *COL28A1* are related to cell to cell adhesion. Of relevance to CRC, *CADM3* encodes a brain-specific Ca-independent protein of the Nectin family that is also involved in cell–cell interactions and has been shown to contribute to metastasis in CRC^49,50^. *L1CAM* is expressed in enteric neurons and is involved in regulating neuronal migration and axon guidance during colon development^51^. It defines the regenerative order of metastasis initiating cells in CRC^52^. The protein encoded by *L1CAM* has been shown to be exclusively detected at the invasive front of CRC tissue; its expression in normal tissue contributes to tumorigenesis and metastasis in CRC cells and its presence on the cell surface makes it a potential therapeutic target^51^. *LUM* is a prognostic oncogene for gastric cancer^53^. Other neuronal proteins have relationships to the colon if not CRC. *PRPH* encodes a cytoskeletal protein found in neurons of the peripheral nervous system and has been associated with neuronal plasticity and axonal regeneration in the colon^54^. *COL14A1* and *COL28A1* encode structural proteins present in the extracellular matrix of the colon and are collagens that contribute to the structural integrity of the colon’s mucosal layer, connective tissues and overall colon function in normal and metastatic colonic tissue^55^. In all, three of the proteins are related to CRC and three others have some role in the colon.

At the lipidomic level, of the 12 classes in the top ten correlated lipid components with the dysplastic factor, TAG was overwhelmingly the most correlated (Fig. 5); six of the ten lipid components were DAG and two were SPH among the other 12 classes (Fig. 5 and Extended Data Fig. 4c). At the metabolomic level, acetyl-carnitine appeared in four of the top 50 metabolite components most correlated with the latent dysplastic factor. Collectively, acetyl-carnitines, TAGs and DAGs are involved in various aspects of colon function and have been studied in relation to colon cancer. For instance, acetyl-carnitine is an acetylated form of the amino acid carnitine, which has a role in energy metabolism^56^, and its supplementation has been investigated as a potential therapeutic target for CRC because of its modulation of cellular metabolisms and potential to enhance anticancer drug (for example, butyrate) sensitivity in SW480 colon cancer cells^57^. Triglycerides are the main storage form of fat in the body that are an important energy source for cells lining the colon (colonocytes)^58^. They also have an important role in lipid metabolism, which is related to CRC progression, and act as a reservoir for FAs used by colonic cells^59^. The aforementioned functional analysis of lipids in M–B and M–D transitions were enriched for the TAG catabolism and metabolism pathway, dominated by downregulated lipids similar to other species related to CRC (for example, CER). Identification of CER as covarying with dysplastic progression reinforces its importance in precancerous colon cancer. Just as TAGs were significant, so were DAGs, which were downregulated (Fig. 4). The importance of DAGs to colon cancer has previously been discussed in the context of PLC signaling and its activation of PKC (usually *PRKCA*), which is associated with colon cancer. However, DAGs and Ca regulate multiple isoforms of PKC; one (*PRKCB2*) is downregulated in colon cancer and was observed as such in the M–D transition in our dataset^60^. Similar to DAGs, SPH and its derivatives such as CER and S1P were previously characterized in this study for the M–D transition and colon cancer and, thus, do not require further elaboration, although the presence of two SHM components among the top ten covarying lipids with the dysplastic factor adds further evidence that this compound and its collaborators are important in precancerous progression.

### Multiomic immune pathway characterization

**Fig. 6:**
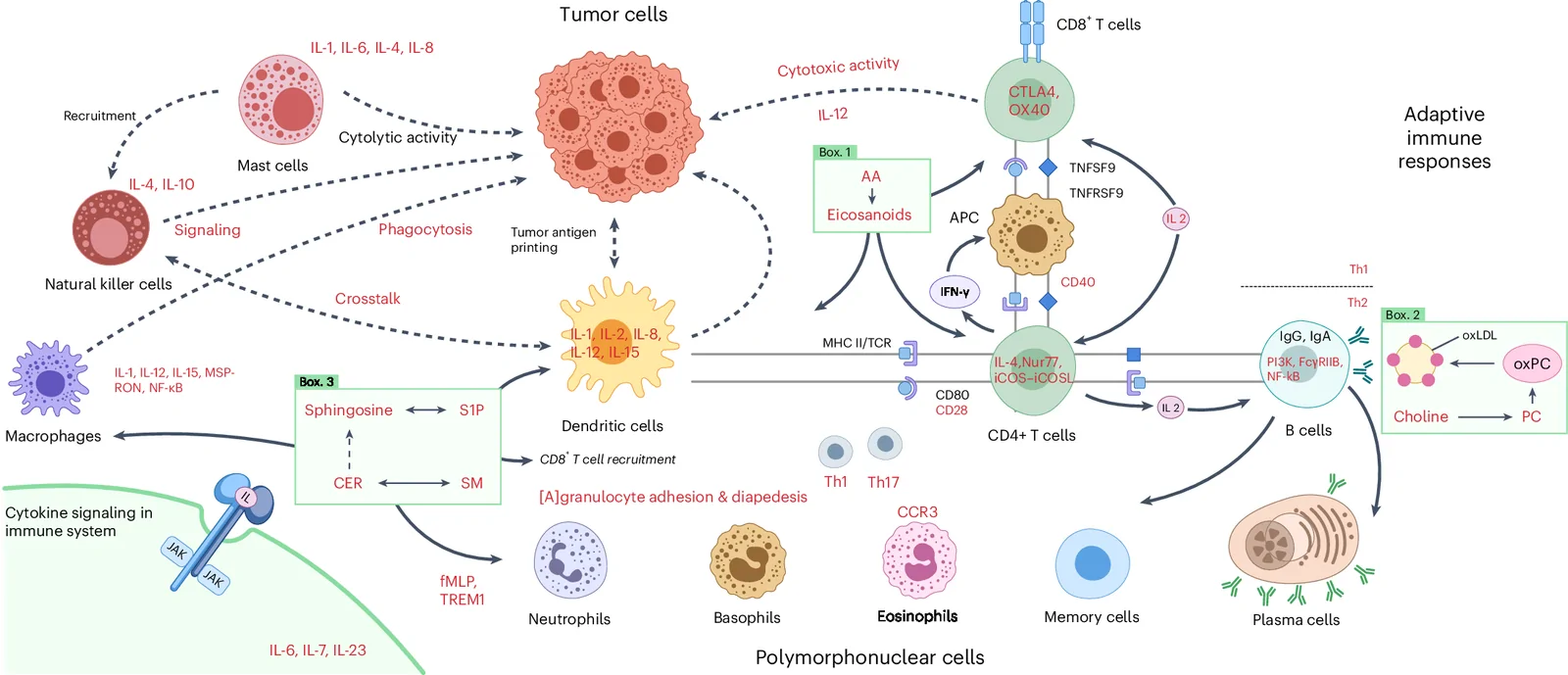
A panorama of immune response pathways in FAP enriched in the M–D transition. The representative immune signaling pathways are marked next to or inside of the symbols of each immune cell type. The immune response pathways that were significantly enriched in our -omic data are highlighted in red. The three boxes contain the key lipid mediators involved in immunity detected in the lipidomic dataset and their chemical relationships. The connections in the map are based on the Qiagen IPA database and literature. Lipidomic and metabolomic connections are based on pathways in CPDB and MSEA enriched for the M–D transition. Portions of this figure were created with BioRender.com. Source data

Given the importance of the immune system in cancer and their extensive changes, we depicted key pathways identified from the multiomic pathway enrichments in the M–D transition (Fig. 6 and Methods). There were 63 proteomic and transcriptomic immune pathways enriched in the M–D transition and the interactions between the cell types in which they are active revealed the extensive network of immune and neuronal cell types that are recruited in the M–D transition. Additionally, the diagram further articulates the multiomic nature of immune system disruption by highlighting lipid and metabolomic immune pathways such as SPs, arachidonic acid and PC, whose downregulation concomitant with SP decrease increases gastric tissue disease such as ulcerative colitis^61^. Moreover, there is significant crosstalk between lipid mediators and cancer and immune cells^62^. However, for CRC, PC and its derived products are usually increased in expression relative to normal tissue. Nevertheless, Fig. 6 details the panoply of diverse cell types, molecular and cellular functions and -omic types that highlight the dynamics of the immune system in the M–D transition.

### B–D transition

Comparison of the B–D transition yielded far fewer molecular and pathway changes than other transitions: 246 molecules (186 metabolites and 60 lipids) and 164 pathways (148 unique at FDR ≤ 5%). Pathway changes were completely characterized by the lipidome. A total of 101 pathways were shared among all transitions; 22 were exclusive to the B–D transition, one was exclusive to the M–B transition (FA metabolism from Reactome) and five were exclusive to the M–D transition. The top six pathways by enrichment score were SP de novo biosynthesis (−log_10_FDR = 7.92), SP metabolism (Reactome, −log_10_FDR = 7.92), MTHFR deficiency (−log_10_FDR = 7.26), signal transduction (−log_10_FDR = 7.26), SP metabolism (Wikipathway, −log_10_FDR = 6.63) and transport of small molecules (−log_10_FDR = 6.30) (Supplementary Table 2). Although the PCA plots did not reveal differences between the polyps, differentially expressed proteins, lipids and metabolites did show up, along with important pathways such as arachidonic acid, ASA, alpha-linolenic acid, linoleic acid and linoleate metabolism, SP biosynthesis and CER signaling, SP metabolism, Ca-dependent events and immune system. These pathways were enriched primarily for downregulated molecules, whereas they were mostly enriched for upregulated molecules in the M–B and M–D transitions. Primarily upregulated molecule enriched pathways for B–D (Fig. 3b) were unique to the transition and consisted of previously mentioned important pathways for CRC: TAG degradation, SP, SPH and S1P metabolism and CER salvage. In general, enrichments in the lipidomic pathways in the B–D transition were reversed in the directionality of the preponderance of the enriched molecules. Given the heavy agreement between M–B and M–D enrichments and directionality, one interpretation may be that those pathways enriched for molecules mostly in a given direction from M–B are then enriched for molecules tending slightly toward the opposite as a compensatory mechanism in precancerous tissue.

## Discussion

We performed a comprehensive multiomic (transcriptome, proteome, metabolome and lipidome) analysis of FAP polyps and adjacent tissues, which represent a valuable model of precancerous progression to CRC. Multiomic analyses revealed numerous molecular and pathway changes in benign polyps relative to mucosa reflecting early changes in early cancer formation (Figs. 2 and 3). Extensive alterations across a number of components in the proteome, lipidome and metabolome were evident; lipidomic and metabolomic changes included arachidonic acid, amino acids, carbohydrates, nucleotides, immune-related lipids and hormones, while proteomic and transcriptomic changes in key pathways were involved in cancer, humoral and cellular immune response, cytokine signaling and cell stress and injury. Many key pathways were involved in cellular homeostasis, metabolism and energy production and organismal survival such as the immune system and were highly relevant to CRC. Key metabolites and lipids included carbohydrates (for example, lactose synthesis), lipids (for example, PC biosynthesis) and amino acids (for example, arginine and proline metabolism).

The transition of polyps to dysplasia was characterized by proteomic and transcriptomic alterations that were predominantly downregulated; functional enrichment was nearly entirely composed of downregulated pathways. Lipid dysregulation in TAG metabolism suggests an energy metabolism shift and putative role for TAGs as an energy source in polyp growth leading to dysplasia. Immune genes were generally downregulated across polyp transitions and the immune response appeared to be activated early during colon hypergrowth, likely reflecting more potent immune surveillance during early disease stages. Arachidonic acid signaling was also upregulated early during polyp formation, contributing to inflammation. While aspirin and NSAIDs are commonly used as prophylactic treatment for persons with FAP, the mechanism of action and optimal timing of this intervention are not known. Of note, we detected significantly decreased expression of key genes in the arachidonic acid signaling pathway. One of them was *PLA2*, encoding phospholipase A2, which converts DAG or phospholipids to arachidonic acid. Another was *ALOX5*, encoding lipoxygenase, which uses arachidonic acid to produce 5-hydroperoxyeicosatetraenoic acid and leukotriene A4, which has an established pathological role in inflammation and infection. On the other hand, increased arachidonic acid signaling molecules were detected at the metabolic level, together with the dysregulation of key genes in the pathway, emphasizing the value of profiling key transitions at multiple molecular scales.

This rich multimodal dataset illuminates the steps involved in precancer formation and transition to malignancy at unprecedented resolution and should serve as a valuable resource for the community. This study is distinct from previous studies on molecular subtypes of heritable or sporadic CRC, as we primarily focused on precancerous tissue as opposed to CRC. FAP polyps are initiated by a distinct mechanism from CRC and arise in persons with Lynch syndrome, which is not molecularly comparable. However, prior studies observed that sporadic CRC can acquire alterations in various components of the Wnt signaling pathway, which is analogous to FAP’s reliance on initiating mutations in *APC*, a key component of the Wnt pathway. Thus, observations in persons with FAP are potentially generalizable to persons with sporadic CRC. Consistent with this, a previous dissection of our FAP biobank using snRNA-seq and ATAC-seq specifically found sporadic samples and FAP samples clustered together by disease stage^9^.

## Methods

### Ethical approval

This study was conducted in compliance with the Stanford Human Research Protection Program guidelines and approved by the Stanford Institutional Review Board (IRB; approval no. 47044). All participants provided written informed consent. Participants consented to have their data from the analyses of their coded samples and coded medical information entered into one of the National Institutes of Health (NIH) databases along with information from the other research participants and used for future research. Only anonymous information from the analyses was deposited to public databases.

### Participant selection

Persons enrolled onto the protocol were screened and selected through the Stanford Cancer Genetics clinic, which provides genetic testing and counseling for persons at risk for hereditary cancer. Potentially eligible participants met one of the following criteria: (1) molecular diagnosis of colorectal polyposis syndrome with a pathogenic or likely pathogenic genetic test result in the *APC* gene and (2) clinical diagnosis of colorectal polyposis syndrome. We also screened and invited persons to participate who were seen through the gastroenterology and hepatology service and adult and pediatric surgery who were undergoing colonoscopy, pouchoscopy or colectomy that met the above criteria. Once a candidate was identified, they were notified of their eligibility to participate in research. Over the phone, the description, risks, benefits and alternatives of participating in the research study were described and a copy of the full consent form was sent to them by email. After giving verbal consent to participate, the clinical research coordinator met with the participant on the same day of the procedure to go over any questions they might have and to sign the consent forms. All participants provided written informed consent.

We did not selectively recruit on the basis of sex, race, or age. The Stanford Cancer Genetics clinic invites persons with FAP from throughout the community and surrounding geographical referral base and invites participation in research studies without discrimination, thus reaching a racially, ethnically diverse population of males and females. We do not set age restrictions in our eligibility requirements.

### Statistics and reproducibility

We discuss in each -omic section the distributional characteristics of each -ome and the appropriateness of the statistical tests used. No statistical method was used to predetermine sample size. No data were excluded from the analyses. The experiments were not randomized. The investigators were not blinded to allocation during experiments and outcome assessment.

### Sample collection

All of the tissues used in this study were procured from colons from total colectomy procedures. The participants’ colons were taken directly from the surgical suite to the Stanford Hospital pathology gross room where they were quickly rinsed and bisected with scissors longitudinally on a room-temperature cutting board typically used in a pathology gross room. Polyp-adjacent normal mucosa (absent any visible polyps) and polyps tissues were carefully dissected from the colon, measured, stored in cryovial tubes and flash-frozen by placing the samples in cryotubes directly into liquid nitrogen. Polyps that had a maximum diameter in any direction that was larger than 10 mm were considered ‘large’, those between 10 mm and 5 mm were ‘medium’ and those less than 5 mm were considered ‘small’. However, these size categories did not apply to normal mucosa. All polyps greater than 10 mm in diameter were frozen-sectioned, stained with hematoxylin and eosin and underwent histopathologic review by project pathologists to assess the percentage of normal versus dysplastic tissue and the presence of dysplasia by grade (low-grade dysplasia versus high-grade dysplasia). Polyps less than 10 mm in size were not uniformly subjected to this histopathologic analysis because of the low likelihood of high-grade dysplasia in this size of polyps (according to the published literature) and the very limited amount of tissue available for analysis. To record the exact location on the colon where the different tissue samples came from for participants A001, A002, A014 and A015, we replaced each tissue sample with a numbered thumbtack and took pictures of the bisected and pinned open colonic lumen. We stitched together the images of the colon, determined the *xy* coordinates of each of the tissue samples in pixel units and then used the diameter of the thumbtacks as a scaling factor to convert pixel units to centimeters. Using a ceramic mortar and pestle, we pulverized the flash-frozen samples in liquid nitrogen to create a fine powder or small chunks as input to each multiomic assay. We isolated RNA using All-Prep kits (Qiagen, 80204). More details can be found at protocols.io (https://www.protocols.io/)^63–65^.

### Transcriptomic analysis

Samples were submitted in three distinct batches: batch 1 to Personalis, batch 2B to Novogene and batch 3 to Novogene. After enforcing an RNA integrity number (RIN) cutoff of 6.0, 21 samples remained from batch 1, 9 remained from batch 2 and 60 remained from batch 3. Samples were processed using the ENCODE RNA-seq uniform peak caller (https://github.com/ENCODE-DCC/rna-seq-pipeline), which uses the following tools: GENCODE^66^ for the reference transcriptome, STARR-seq^67^ for alignment and RSEM^68^ for gene quantitation, with estimated counts summarized at the gene level. Lack of replicates across batches (for example, in batches 1 and 3) and material differences in technical factors (for example, library preparation) between Personalis and Novogene submissions made adjustments for batch effects too precarious, even with latent variable methods. As our data consisted of count data, we opted for a negative binomial model for modeling and Wald tests for hypothesis tests. Thus, differential expression analysis using DESeq2 (R package version 1.40.2)^69^ proceeded within each (participant, batch and contrast) triplicate separately for any contrast (transition) for which there were at least two samples for each of the precancerous stages being contrasted; no additional variables were adjusted given this restricted design. These analyses yielded the M–A plots in Extended Data Fig. 2, which also shows the total number of samples (61) and unique samples when not considering the batch (60) that factored into the analysis. Differentially expressed transcripts were called at FDR ≤ 5% and fold change (FC) of ±15% for each participant, batch and contrast. Summarizing to transitions and contrasts across multiple participants and batches eliminated the reporting of differential transcripts for certain transitions. For B–D, analyses yielding Extended Data Fig. 2a,e both produced differential transcripts; however, there was no agreement among differential transcripts between participants A001 and F and, as a result, we reported no differential transcripts for this transition. For M–B, the analysis yielding Extended Data Fig. 2a produced differential transcripts but only for a single participant or batch and, as with B–D, we reported no differential transcripts for this transition. We did report differential transcripts for the M–D transition. Differential transcripts for M–D were sourced from the M–D comparisons that produced Extended Data Fig. 2a–c (differential transcripts corresponding to the analysis that produced Extended Data Fig. 2d were omitted because of their low numeracy); differential transcripts that agreed on directionality in two of the three analyses were reported as differential for the M–D transition. Thus, differential transcripts with FDR ≤ 5% and that were at least 15% increasing or decreasing were reported only for the M–D transition. More details can be found at protocols.io (https://www.protocols.io/)^70,71^.

### Proteomic analysis

#### Sample preparation

Flash-frozen tissue samples were smashed into small pieces in the mortar and pestle and further disrupted using beat beating and sonication in lysis buffer (6 M guanidine, 10 mM TCEP, 40 mM CAA and 100 mM Tris pH 8.5). The supernatant was collected and heated at 95 °C for 5 min. After protein reduction and alkylation, protein concentration was measured using a BCA kit (Thermo Fisher Scientific). Protein extract was digested using LysC (1:100 protease-to-protein ratio) for 2 h at room temperature followed by trypsin (1:50) digestion overnight at 37 °C. Peptides were cleaned up using a Waters HLB column and subsequently labeled using TMT10Plex (Thermo Fisher Scientific) in 100 mM TEAB buffer according to the manufacturer’s recommendations. An equal amount of protein from each tissue was pooled together as a reference sample. More details can be found at protocols.io (https://www.protocols.io/)^72^.

### TMT experimental design

In this study, we used TMT10plex, which can label up to ten samples in one experiment. We randomized tissue samples such that each TMT10plex consisted of an assortment of tissues. To facilitate cross-tissue comparison and reduce the technical variation among MS runs, pooled reference samples were added into each TMT10plex experiment. Equal amounts of nine samples and common reference samples were multiplexed into one TMT10plex run. We also performed two technical replicates for all samples. In the replicate, samples had the same TMT labels as in the initial run but they were rerandomized to mix with different samples in each TMT10plex run.

### Proteomic data normalization

Proteomic data were acquired using the TMT quantitation approach. We pooled all the samples together and used it as a reference sample. The same amount of reference sample was added to each run. One of the main advantages of TMT-based quantitative methods is that we can use a common reference sample to normalize our data^73^. We did not use the median normalization method. In each multiplexed run, we manually checked to make sure there was no overrepresentation of any kind of sample. Before each run, we also checked the ratio of each sample to make sure it was mixed in a 1:1 ratio.

### Two-dimensional (2D) LC separation

We used the Waters online nano 2D LC system for fractionation using approximately 15 μg of multiplexed sample. Peptides were separated by RPLC at high pH in the first dimension, followed by an orthogonal separation at low pH in the second dimension. In the first dimension, the mobile phases were buffer A (20 mM ammonium formate at pH 10) and buffer B (acetonitrile). Peptides were separated on an Xbridge C18 5.0-μm column (300 μm × 5 cm; Waters) using 12 discontinuous steps of buffer B at 2 μl min^−1^ flow rate. In the second dimension, peptides were loaded to an in-house packed 25-cm Sepax GP-C18 1.8-μm resin column (inner diameter, 75 μm; tip, inner diameter, 15 μm tip) with buffer A (0.1% formic acid in water). Peptides were separated with a linear gradient from 5% to 30% buffer B (0.1% formic acid in acetonitrile) at a flow rate of 600 nl min^−1^ in 180 min. The LC system was directly coupled inline with an Orbitrap Fusion Lumos (Thermo Fisher Scientific).

### MS data acquisition and analysis

The Orbitrap Fusion was operated in data-dependent mode for both MS2 and MS3. The MS1 scan was acquired in the Orbitrap mass analyzer with a resolution of 120,000 at *m*/*z* 400. The top-speed instrument method was used for MS2 and MS3. For MS2, the isolation width was set at 0.7 Da and isolated precursors were fragmented by collision-induced dissociation at a normalized collision energy (NCE) of 35% and analyzed in the ion trap using ‘turboscan’. Following the acquisition of each MS2 spectrum, a synchronous precursor selection (SPS) MS3 scan was collected on the top five most intense ions in the MS2 spectrum. SPS MS3 precursors were fragmented by higher-energy collision-induced dissociation at an NCE of 65% and analyzed using the Orbitrap at a resolution of 60,000. Each sample was run again on another Orbitrap Fusion in the same lab with the exact same settings for technique replicates.

We used SEQUEST in ProteomeDiscoverer 2.1 (Thermo Fisher Scientific) for protein identification. Raw files from 12 fractions of each sample were combined together for a single search against the UniProt human proteome database. A mass tolerance of 10 ppm and 0.6 Da was used for precursor ions and fragment ions, respectively. The search included cysteine carbamidomethylation as a fixed modification. Peptide N-terminal and lysine TMT10plex modification, protein N-terminal acetylation and methionine oxidation were set as variable modifications. Up to two missed cleavages were allowed for trypsin digestion. The peptide FDR was set as <1% using Percolator. For protein identification, at least one unique peptide with a minimum length of six amino acids was required. For protein quantitation, only unique peptides with a precursor ion isolation purity > 50% were used. Peptides passing the criteria were summed to represent protein abundance.

### Differential analysis

A linear mixed model was used to predict protein abundance from sample stage while adjusting for fixed effects (participants’ race, sex and age and sample location) and a single random effect intercept (participant). The R package nlme (version 3.1.162) was used and separate variances were estimated for each sample stage^74^. Contrasts were performed for M–B, M–D and B–D and proteins with an FC of ±15% and FDR ≤ 1% were called differentially abundant. Analysis of variance (ANOVA; type III) was used and a one-sided *F*-test was used with an *F*-statistic for hypothesis testing. The normality of data was checked graphically.

### Untargeted metabolomic and targeted lipidomic analysis

#### Sample preparation

Comprehensive profiling of complex lipids and metabolites was performed on 77 colon tissue samples from six persons. Roughly 30 mg of flash-frozen tissue was homogenized in 500 µl of ice-cold methanol by bead beating (MP Bioscience, 6913-100) at 4 °C (2 × 45 s). Metabolites and complex lipids were extracted using a biphasic separation with cold MTBE, methanol and water. Briefly, 1 ml of ice-cold MTBE was added to 300 μl of the homogenate spiked in with 40 µl of deuterated lipid internal standards (Sciex, 5040156, lot no. LPISTDKIT-102). The samples were then sonicated (3 × 30 s) and agitated at 4 °C for 30 min. After the addition of 250 μl of ice-cold water, the samples were vortexed for 1 min and centrifuged at 14,000*g* for 5 min at 20°C. The upper organic phase contained the lipids, the lower aqueous phase contained the metabolites and the proteins were precipitated at the bottom of the tube. For quality control (QC), three reference plasma samples (40 µl of plasma), two normal colon tissue samples, one colonic polyp sample and one control lacking any sample (blank) were processed in parallel. More details can be found at protocols.io (https://www.protocols.io/)^75–78^.

1. **Metabolites**: Proteins were further precipitated by adding 500 μl of 33:33:33 acetone, acetonitrile and methanol spiked in with 15 labeled metabolite internal standards to 300 μl of the aqueous phase and 200 μl of the lipid phase and incubating the samples overnight at −20 °C. After centrifugation at 17,000*g* for 10 min at 4 °C, the metabolic extracts were dried down to completion and resuspended in 100 μl of 50:50 methanol and water.
2. **Complex lipids**: First, 700 µl of the organic phase was dried down under a stream of nitrogen and resolubilized in 200 μl of methanol for storage at −20 °C until analysis. The day of the analysis, samples were dried down, resuspended in 300 μl of 10 mM ammonium acetate in 90:10 methanol and toluene and centrifuged at 16,000*g* for 5 min at 24 °C.

### Data acquisition

Metabolite extracts were analyzed using a broad-spectrum untargeted LC–MS platform as previously described^8^, while complex lipids were quantified using a targeted MS-based approach^79^.

1. **Untargeted metabolomics by LC–MS**. Metabolic extracts were analyzed four times using HILIC and RPLC separation in both positive and negative ionization modes. Data were acquired on a Thermo Q Exactive HF MS instrument for HILIC (Thermo Fisher Scientific) and a Thermo Q Exactive MS instrument for RPLC (Thermo Fisher Scientific). Both instruments were equipped with an HESI-II probe and operated in full MS scan mode. MS/MS data were acquired on QC samples consisting of an equimolar mixture of all samples in the study. HILIC experiments were performed using a ZIC-HILIC column (2.1 × 100 mm, 3.5 μm, 200 Å; Merck Millipore) and mobile-phase solvents consisting of 10 mM ammonium acetate in 50:50 acetonitrile and water (A) and 10 mM ammonium acetate in 95:5 acetonitrile and water (B). RPLC experiments were performed using a Zorbax SBaq column (2.1 × 50 mm, 1.7 μm, 100 Å; Agilent Technologies) and mobile-phase solvents consisting of 0.06% acetic acid in water (A) and 0.06% acetic acid in methanol (B). Data quality was ensured by (1) injecting 6 and 12 pool samples to equilibrate the LC–MS system before running the sequence for RPLC and HILIC, respectively; (2) injecting a pool sample every ten injections to control for signal deviation with time; and (3) checking mass accuracy, retention time and peak shape of internal standards in each sample.
2. **Targeted lipidomics using the Lipidyzer platform**. Lipid extracts were analyzed using the Lipidyzer platform that comprises a 5500 QTRAP system equipped with a SelexION differential mobility spectrometry (DMS) interface (Sciex) and a high-flow LC-30AD solvent delivery unit (Shimazdu). Briefly, lipid molecular species were identified and quantified using multiple reaction monitoring (MRM) and positive and negative ionization switching. Two acquisition methods were used covering 12 lipid classes; method 1 had SelexION voltages turned on while method 2 had SelexION voltages turned off. Data quality was ensured by (1) tuning the DMS compensation voltages using a set of lipid standards (Sciex, 5040141) after each cleaning, more than 24 h of idling or 3 days of consecutive use; (2) performing a quick system suitability test (Sciex, 5040407) before each batch to ensure an acceptable limit of detection for each lipid class; and (3) triplicate injection of lipids extracted from a reference plasma sample (Sciex, 4386703) at the beginning of the batch.

### Data processing

Data were acquired in two separate batches and the batch effect was controlled by running three samples in common in both batches.

1. **Metabolomics**. Data from each mode were independently analyzed using Progenesis QI software (version 2.3; Nonlinear Dynamics). Metabolic features from blanks and those that did not show sufficient linearity upon dilution in QC samples (*r* < 0.6) were discarded. Only metabolic features present in more than two thirds of the samples were kept for further analysis. Median normalization was applied to correct for differential starting material quantity. Missing values were inputted by drawing from a random distribution of low values in the corresponding sample. The batch effect was corrected by the ComBat model using the dbnorm package^80,81^ Data quality after normalization was verified by ensuring clustering of three biological replicates analyzed in two batches on a PCA plot (Extended Data Fig. 1). Data from each mode were merged and 10,520 metabolic features were annotated. Peak annotation was first performed by matching experimental *m*/*z*, retention time and MS/MS spectra to an in-house library of analytical-grade standards. Remaining peaks were identified by matching experimental *m*/*z* and fragmentation spectra to publicly available databases including the Human Metabolome Database (HMDB), MassBank of North America and MassBank using the R package ‘metID’ (version 0.2.0). Metabolites were reported if the similarity score was above 0.65. We used the Metabolomics Standards Initiative level of confidence to grade metabolite annotation confidence (level 1–3). Level 1 represents formal identifications, where the biological signal matches accurate mass, retention time and fragmentation spectra of an authentic standard run on the same platform. For level 2 identification, the biological signal matches accurate mass and fragmentation spectra available in one of the public databases listed above. Level 3 represents putative identifications that are the most likely name on the basis of previous knowledge. In total, 1,157 metabolites were identified and their abundances were reported as spectral counts.
2. **Targeted lipidomics***.* Lipidyzer data were reported by the Lipidomics Workflow Manager (version 1.0.5.0) software, which calculates concentrations for each detected lipid as the average intensity of the analyte MRM divided by the average intensity of the most structurally similar internal standard MRM multiplied by its concentration. Lipids detected in less than two thirds of the samples were discarded and missing values were inputted by drawing from a random distribution of low values classwise in the corresponding sample. Median normalization (excluding TAG and DAG) was applied to correct for differential starting material quantity. Batch normalization was performed using QC reference plasma samples run in both batches. Data quality after normalization was verified by ensuring the clustering of three biological replicates analyzed in two batches on a PCA plot (Extended Data Fig. 1). We detected 514 individual lipid species belonging to 12 classes (CE, CER, DAG, free FA, hexosyl CER, lactosyl CER, LPE, LPC, PC, PE, SPH and TAG) and their abundance was reported as the concentration in nmol g^−1^.

### Differential analysis

Differential analysis was performed on 77 samples from six persons. For each contrast across stages and each metabolite or lipid species, a binary logistic model was designed to predict the stage with higher dysplasia (encoded with 1) from the species’ log_2_ abundance, adjusting for both fixed effects (sex and age) and random effects (participant identifier). This model was implemented and run in R using glmer from the lme4 R package (version 1.1.33) with parameters verbose = 2, nAGQ = 9 and family = binomial, and a bobyqa optimizer to control parameters with a maximum of 100,000 iterations. For each contrast, models were trained only on samples in stages relevant to the contrast. Both single (including all variables) and ANOVA models (including and excluding metabolites and lipids) were applied. Applying stringent criteria, the highest detected *P* value per hit was considered as the level of significance. Data were log_2_-transformed before analysis and, thus, the estimated coefficient per variable is the log_2_FC weighted for mean variance. For consistency with other -omic data, the estimated coefficient of metabolic feature (metabolites or lipids) is reported as the log_2_FC. Before applying statistical models, we evaluated the distribution and normality of the data using graphical methods and statistical tests. Briefly, in our study of metabolomic and lipidomic data, we randomly selected features or species to check for normality using the Shapiro–Wilk test and graphical methods, including density plots and residual plots. Features or species that met the normality assumptions were then included in the loop for differential analysis.

### Functional pathway enrichment analysis

To produce the multiomic pathway enrichments in Supplementary Table 2, one set of tools was used for the transcriptome and proteome and another set was used for the lipidome and metabolome. For the proteomic and transcriptomic data, the IPA (Qiagen; https://www.qiagenbioinformatics.com/products/ingenuitypathway-analysis) platform was used to conduct pathway enrichment analysis, enriching for differentially expressed genes (Supplementary Table 1a; more details can be found in the section on transcriptomic differential analysis) and circulating proteins (Supplementary Table 1b; more details can be found in the section on proteomic differential analysis). Significance levels of pathways were determined by the hypergeometric test (one-sided) in IPA and *P* values were corrected by the Benjamini–Hochberg procedure. Differential pathways were called for the M–B, M–D and B–D transitions at FDR ≤ 5%. Enrichments were performed with downregulated and upregulated molecules separately and the directionality of the molecules in the enriched set informed the pathway’s directional regulation (that is, pathways enriched for downregulated molecules were deemed downregulated and the same applied for upregulated pathways and molecules). To determine functional mechanisms behind the significant changes in the lipidome (Supplementary Table 1c; more details can be found in the section on lipidomic differential analysis) and metabolome (Supplementary Table 1d; more details can be found in the section on metabolomic differential analysis), we performed CPDB^11^ ORA^12^ and MSEA^13^, respectively. Functional annotations were performed against databases such as KEGG, Small Molecule Pathway Database, BioCarta and HumanCys. The obtained *P* values from enrichment analysis were corrected for multiple hypotheses using the Benjamini–Hochberg method and pathways with FDR ≤ 5% were considered significant. Enrichments were performed with all differential molecules (downregulated and upregulated molecules together, not separated) and pathways were characterized as downregulated and upregulated according to the median log_2_FC of the enriched molecules.

### Multiomic integration analysis at pathway level

To construct the dot plot and heat map of notable pathway enrichments in Fig. 3, significantly enriched pathways from different -omes were curated and investigated systematically, checking across both transitions and -omes. On the basis of the functionality and diversity of enriched transcriptomic and proteomic pathways, important transcriptomic and proteomic pathways were identified and classified into one of the following categories (Fig. 3a): (1) nuclear receptor signaling; (2) lipid biosynthesis; (3) organismal growth and development; (4) cancer; (5) intracellular signaling; (6) cellular stress and injury; (7) disease-specific pathways; (8) cellular immune response; (9) humoral immune response; (10) cellular growth and development; and (11) cytokine signaling. The same procedure was repeated for the lipidomic and metabolomic pathways; key pathways were identified and classified into the following categories (Fig. 3b): (1) amino acids; (2) carbohydrates; (3) energy; (4) immune; (5) lipids; (6) lipids and immune mediators; (7) neurotransmitter metabolism; and (8) nucleotides. Pathway directions were calculated using the median FC values for the significant molecules in the pathway.

### Multiomic network construction

The multiomic network diagram of Fig. 4 was constructed by examination of which proteomic or transcriptomic IPA pathways highly enriched in the M–D transition intersected with important differential metabolites and lipids (indicated by KEGG or HMDB identifiers). We selected four major pathways enriched in the proteome or transcriptome for M–D (that is, PLC signaling, FXR and RXR activation, IL-12 signaling and production and S1P signaling) and major pathways enriched in the lipidome and metabolome (SP metabolism, SPH metabolism and CER signaling). These pathways were plotted along with their constitutive members in the differential transcriptomic, proteomic, metabolomic and lipidomic datasets; lines were drawn from pathways to molecules to indicate membership and differential molecules were colored according to their log_2_FC (or median log_2_FC if multiple molecules were collapsed). This network construction shows the intersection among these highly enriched pathways across multiple -omes. More details can found in the Fig. 4 caption.

### Multiomic factor analysis (MOFA)

The MOFA^82^ seen in Fig. 5 and Extended Data Figs. 3,4 was conducted as follows. First, a thorough examination was carried out to assess the normal distribution of all -omic data. As a result of this evaluation, the lipidomic and transcriptomic count data were subjected to a log_2_ transformation. Subsequently, a comprehensive analysis was performed using MOFA through the MOFA2 R package (version 1.10.0) to amalgamate data originating from diverse -omic sources, encompassing metabolomics, lipidomics, transcriptomics and proteomics. In the course of this analysis, the identification of a latent factor, the first latent factor (factor 1) emerged. Factor 1 was found to be notably influenced by variants linked to conditions such as dysplasia. To elucidate the common patterns or biological associations encapsulated by factor 1, a visualization was generated. This visualization showcases the top 100 features from each -omic dataset, organized on the basis of their respective factor 1 values. Notably, within the lipidomic data, TAG species demonstrated a significant influence on the entire factor 1. To distill this impact, an average value was computed for all TAG species exhibiting a factor value > 0.3. This aggregated value was subsequently plotted for visualization. It is important to note that the factor values are presented as weighted values scaled to their absolute values. This adjustment was made to address the variations in scale among different data sources. Because the measurement scales could differ significantly among sources, the weights assigned to each view or variable were not directly comparable in their original units. Scaling them to their absolute values helped to standardize the contributions of these variables, making them more comparable and interpretable when integrated into the analysis.

### Multiomic landscape of immune response pathways

To construct Fig. 6, the significantly enriched pathways related to immune response were extracted and examined. The representative top significant immune response pathways were highlighted within each immune cell type. The connections were built according to the IPA database and literature mining. The key lipid mediators in immunity were highlighted in the boxes. The illustration of the landscape of immune response pathways was conducted using BioRender.com.

### Batch design

For the transcriptome, samples were submitted in three distinct batches: batch 1 to Personalis, batch 2B to Novogene and batch 3 to Novogene. After enforcing an RIN cutoff of 6.0, 21 samples remained from batch 1, 9 remained from batch 2 and 60 remained from batch 3. Differential expression analysis proceeded within each batch and participant such that batch effects were controlled explicitly in the statistical design. For the proteome, samples were processed in two distinct batches and normalized relative to a common reference of pooled samples; differential expression analysis controlled for batch effect in the statistical design. For the lipidome and metabolome, samples were run in two batches with three biological replicates in common. The batch effect was corrected by the ComBat model using the dbnorm package^80,81^ and quality after normalization was verified by ensuring the clustering of three biological replicates analyzed in two batches on a PCA plot.

### Participant-specific analysis

For the proteome, lipidome and metabolome, the linear mixed model analysis was repeated within each participant, removing the participant as a blocking factor. For the transcriptome, DESeq2 was run within each participant and batch. In Supplementary Table 4a, we report the number of samples by participant and batch for each -ome. We report the participant-specific differential molecules in the transcriptome, proteome, lipidome and metabolome in Supplementary Table 5a–d. We discuss the participant-specific analysis by -omic below.

For the transcriptome, the reported number of differentially expressed molecules arose from participant-specific statistics. The differential analysis was performed within each participant and batch and aggregated (Extended Data Fig. 4). For the M–B transition, only one participant admitted sufficient samples for differential analysis and, thus, they were omitted. For the B–D transition, there was very little agreement between the number of differentially expressed transcripts in A001 and F; thus, these differential transcripts were not aggregated and reported. For the M–D transition, differential transcripts common to either batch of A002 samples (batch 1 and 3) and A001 (batch 1) were reported as differentially expressed (A015 was excluded for the low number of differential transcripts). The differential transcripts were reported if they were differentially expressed at |FC| > 1.15 (or |log_2_FC| ≥ 0.321928095 and FDR ≤ 5% in two of the three contrasts with the same directionality (the median log_2_FC and FDR are computed and reported). This resulted in 2,110 transcripts reported as differentially expressed for the M–D transition and 0 for the M–B and B–D transitions. Supplementary Table 4a incorporates the number of transcripts detected for each participant and batch, as well as the number of samples that contributed to each analysis. This is congruent with the results reported in Extended Data Fig. 4. We report these participant-specific and batch-specific differential transcripts in Supplementary Table 5a.

In Supplementary Table 4b, we report the intersection of differential transcripts for each participant and batch. Note that because only one participant is reported for M–B and there is very little overlap between the differential transcripts for F.3 and A001.1 for B–D, no differential transcripts are reported for F and A001 for M–B or M–D.

We also compared participant-specific differential transcripts to those aggregated across all participants (Supplementary Table 4c). There was high concordance between the globally inferred M–D differential transcripts and those specific to A001.1, A002.1 and A002.3, which was expected because these participant-specific transcripts were aggregated to produce the globally inferred M–D differential transcripts.

For the proteome, we replicated the whole-cohort analysis for each participant individually (removing it as a blocking factor in the linear mixed model). In Supplementary Table 4a, we report the number of differential proteins at FDR ≤ 1% with |log_2_FC| ≥ log_2_(1.5) = 0.58. In Supplementary Table 4d, we expand this to all pairwise intersections of differential proteins at FDR ≤ 1% with |log_2_FC| ≥ 0.58. There was no overlap among all M–D participant analyses. We report the differential proteins from the individual level analysis in Supplementary Table 5b.

We intersected each participant-specific differential protein set with the differential proteins inferred across all participants (Supplementary Table 4e). For participant A002 in the M–D transition, 386 and 621 of 838 differential proteins overlapped with the 525 and 926 differential proteins inferred across all participants in the M–B and M–D transitions, respectively. For participant F in M–B, three of four differential proteins were replicated in the M–B and M–D transitions; for participant F in the M–D transition, 8 of 11 differential proteins were also differentially expressed across all participants in M–B and M–D. For participant G, five of nine differential proteins in the M–D transition were differentially expressed in M–B and M–D. This indicates that participant-specific proteins were largely captured in the reported differential proteins across all participants.

For the metabolome and lipidome, we report the number of samples available for each participant and stage and the number of differential lipids and metabolites detected in Supplementary Table 4a at a nominal *P* value of 0.05. None of the metabolites intersected across participants and transitions at the individual level. We report the differential lipids and metabolites in Supplementary Table 4c,d.

We detected slightly more B–D differential lipids in the participant-specific analysis for F than across all participants (122 and 60, respectively). Of the 122 participant-specific lipids, 99 were downregulated and 23 were upregulated. The overlap when filtering by a *P* value of 0.05 and ensuring the coefficient was consistent by sign in both analyses was 20, 34 and 42 between the differential lipids from participant F in B–D and the differential lipids across all participants in M–B, M–D and B–D, respectively. The total number of differential lipids for these transitions was 402, 438 and 60, respectively, with a fairly low concordance of the participant-specific differential lipids and those inferred across all participants (Supplementary Table 4f).

We repeated this comparison in the metabolome (Supplementary Table 4g). For participant F in the M–D transition, the only significant metabolite was downregulated and replicated (*P* value ≤ 0.05 and consistent sign of coefficient) in the M–B and M–D transitions across all participants but not the B–D transition. For participant F in the B–D transition, we detected slightly more differential metabolites (157) than across all participants in the B–D transition (95); of the 157, 66 were downregulated, 91 were upregulated and 3, 29 and 39 were replicated across all participants in the M–B, M–D and B–D transitions, respectively. The numbers of differential metabolites across all participants for the M–B, M–D and B–D transitions were 158, 223 and 95, respectively, and this overlapped at a low rate with the differential metabolites inferred across participants. For participant G in the B–D transition, only one of the two upregulated metabolites was replicated in the M–D transition.

Overall, we observed that subsetting the data by individual decreased the number of reported molecules in the proteome slightly for M–D and critically for M–B; there were marginally more detected metabolites in the lipidome and metabolome. In the transcriptome, the individual analysis was a component of the joint analysis; however, Supplementary Table 4a gives more insight into the rationale behind our differential expression calling (that is, only one participant for M–B and lack of concordance among B–D participant-level differential transcripts).

### Reporting summary

Further information on research design is available in the Nature Portfolio Reporting Summary linked to this article.

## Supplementary information

Reporting Summary

Supplementary Tables Supplementary Tables 1–5.

## Source data

Source Data Fig. 1 Cohort data.

Source Data Fig. 2 Differential expression statistics.

Source Data Fig. 3 Pathway enrichment statistics.

Source Data Fig. 4 Molecules with log_2_FC and FDR statistics.

Source Data Fig. 5 MOFA factor 1 statistics.

Source Data Fig. 6 Pathway enrichment statistics.

## Data Availability

All the data for this study were deposited to the HTAN data portal (https://humantumoratlas.org/explore) through the Synapse platform alongside the corresponding metadata for the assays, donors and biospecimens. Dataset identifiers and uniform resource locators are provided (RNA-seq in Supplementary Table 3a, proteomics in Supplementary Table 3b, lipidomics in Supplementary Table 3c and metabolomics in Supplementary Table 3d). MS data are available on Synapse after registration. RNA-seq datasets are shared through the Seven Bridges Cancer Genomics Cloud and access is controlled through the NIH database of Genotypes and Phenotypes (accession number phs002371) to protect patient privacy as per the IRB-approved data sharing plan and HTAN standard procedures^83^. Access to the RNA-seq data can be requested by principal investigators and requires the submission of a data use certification agreement to the NCI’s Data Access Committee (NCIDAC@mail.nih.gov). Source data are provided with this paper.

## Code availability

Source code consisted of the straightforward application of statistical libraries in R and is available on request (mpsnyder@stanford.edu).
