# Supplementary material for: Multiomic analysis of familial adenomatous polyposis reveals molecular pathways associated with early tumorigenesis
Source: Nat Cancer. 2024 Oct 30;5(11):1737–53. doi: 10.1038/s43018-024-00831-z (PMC11584401; doi:10.1038/s43018-024-00831-z)
Supplement: Supplementary file 1 — Reporting Summary [file 43018_2024_831_MOESM1_ESM.pdf]

nature portfolio

Corresponding author(s): Mike Snyder

Last updated by author(s): 8/2/2024

Reporting Summary

Nature Portfolio wishes to improve the reproducibility of the work that we publish. This form provides structure for consistency and transparency in reporting. For further information on Nature Portfolio policies, see our Editorial Policies and the Editorial Policy Checklist.

Statistics

For all statistical analyses, confirm that the following items are present in the figure legend, table legend, main text, or Methods section.

n/a	Confirmed	
☐	☒	The exact sample size (*n*) for each experimental group/condition, given as a discrete number and unit of measurement
☐	☒	A statement on whether measurements were taken from distinct samples or whether the same sample was measured repeatedly
☐	☒	The statistical test(s) used AND whether they are one- or two-sided<br>*Only common tests should be described solely by name; describe more complex techniques in the Methods section.*
☐	☒	A description of all covariates tested
☐	☒	A description of any assumptions or corrections, such as tests of normality and adjustment for multiple comparisons
☒	☐	A full description of the statistical parameters including central tendency (e.g. means) or other basic estimates (e.g. regression coefficient) AND variation (e.g. standard deviation) or associated estimates of uncertainty (e.g. confidence intervals)
☐	☒	For null hypothesis testing, the test statistic (e.g. *F*, *t*, *r*) with confidence intervals, effect sizes, degrees of freedom and *P* value noted<br>*Give P values as exact values whenever suitable.*
☒	☐	For Bayesian analysis, information on the choice of priors and Markov chain Monte Carlo settings
☒	☐	For hierarchical and complex designs, identification of the appropriate level for tests and full reporting of outcomes
☐	☒	Estimates of effect sizes (e.g. Cohen's *d*, Pearson's *r*), indicating how they were calculated

Our web collection on statistics for biologists contains articles on many of the points above.

Software and code

Policy information about availability of computer code

Data collection	Sequest Proteome Explorer (v2.1) was used for proteomic data annotation and normalization. Metabolomics processing used Progenesis QI (v2.3) for data processing of each mode. Lipidomics data was reported by the Lipidomics Workflow Manager (LWM, v 1.0.5.0). Transcriptomic data was processed using the ENCODE Uniform Peak Processing Pipeline (https://github.com/ENCODE-DCC/rna-seq-pipeline).
Data analysis	Transcriptomic data was subjected to differential analysis using the DESeq2 R package (v1.40.2). Proteomic data was subjected to differential analysis using linear mixed model's via the nlme R package (3.1.162). Metabolites were identified using the metID R package (v0.2.0). Metabolites and lipids were subjected to differential analysis using glmer via the lme4 R package (1.1.33). Pathway analysis was performed for transcriptomic/proteomic data using Qiagen Ingenuity Pathway Analysis (IPA) and for lipidomic/metabolomic data using ConsensusPathDB and Metabolite Set Enrichment Analysis (MSEA) respectively via webtools. Multiomic Factor Analysis was performed using the MOFA2 R package (v1.10.0).<br><br>Source code consists of straightforward application of statistical libraries in R and is available on request: mpsnyder@stanford.edu

For manuscripts utilizing custom algorithms or software that are central to the research but not yet described in published literature, software must be made available to editors and reviewers. We strongly encourage code deposition in a community repository (e.g. GitHub). See the Nature Portfolio guidelines for submitting code & software for further information.

Data

Policy information about availability of data

All manuscripts must include a data availability statement. This statement should provide the following information, where applicable:

- Accession codes, unique identifiers, or web links for publicly available datasets
- A description of any restrictions on data availability
- For clinical datasets or third party data, please ensure that the statement adheres to our policy

All the data for this study have been deposited through the Human Tumor Atlas Network (HTAN) data portal (https://humantumoratlas.org/explore) via the Synapse platform alongside the corresponding metadata for the assays, donors, and biospecimens. Dataset IDs and URLs are provided (RNA-seq in Supplementary Table 3A, proteomics in Supplementary Table 3B, lipidomics in Supplementary Table 3C, metabolomics in Supplementary Table 3D). Mass spectrometry data are available on Synapse after registration. RNA-sequencing datasets are shared through the Seven Bridges Cancer Genomics Cloud and access is controlled through the NIH database of Genotypes and Phenotypes (dbGaP, Study Accession phs002371) to protect patient privacy per the IRB-approved data sharing plan and HTAN standard procedures (de Bruijn et al. 2024). Access to the RNA-sequencing data can be requested by principal investigators and requires the submission of a Data Use Certification Agreement to the National Cancer Institute's Data Access Committee (NCIDAC@mail.nih.gov).

Research involving human participants, their data, or biological material

Policy information about studies with human participants or human data. See also policy information about sex, gender (identity/presentation), and sexual orientation and race, ethnicity and racism.

Reporting on sex and gender	We do not report any sex or gender specific effects. We control for them in certain regression analyses.
Reporting on race, ethnicity, or other socially relevant groupings	We do not comment on ethnic specific effects. We control for them in certain regression analyses.
Population characteristics	The population consists of six individuals from Hispanic or Caucasian ancestry. We report demographic characteristics in Table 1 of our study.
Recruitment	Patients enrolled onto the protocol were screened and selected through the Stanford Cancer Genetics clinic, which provides genetic testing and counseling for persons at risk for hereditary cancer. Potentially eligible participants met one of the following criteria: 1). Molecular diagnosis of colorectal polyposis syndrome with a pathogenic/likely pathogenic genetic test results in the APC gene 2). Clinical diagnosis of colorectal polyposis syndrome. We also screened and invited patients to participate who were seen through the Gastroenterology and Hepatology service and Adult and Pediatric Surgery who were undergoing colonoscopy, pouchoscopy or colectomy that met the above criteria. Once a patient was identified, they were notified of their eligibility to participate in research. Over the phone, the description, risks, benefits, and alternatives of participating in the research study are described and a copy of the full consent form was sent to them via email. After giving verbal consent to participate, the clinical research coordinator met with the patient on the same day of the procedure to go over any questions they might have and to sign the consent forms. All patients provided written informed consent to participate.<br>We did not selectively recruit based on sex, race, or age. The Stanford Cancer Genetics clinic invites FAP patients from throughout the community and surrounding geographical referral base, and invites participation in research studies without discrimination, thus reaching a racially, ethnically diverse population of males and females. We do not set age restrictions in our eligibility requirements.<br>Further information on research design is available in the Nature Research Reporting Summary linked to this article.
Ethics oversight	This study was conducted in compliance with the Stanford Human Research Protection Program guidelines and approved by the Stanford Institutional Review Board (IRB# 47044). All participants provided written informed consent. Participants consented to have their data from the analyses of their coded samples and coded medical information entered into one of the National Institutes of Health (NIH) databases along with information from the other research participants and used for future research. Only anonymous information from the analyses was deposited in public databases.

Note that full information on the approval of the study protocol must also be provided in the manuscript.

Field-specific reporting

Please select the one below that is the best fit for your research. If you are not sure, read the appropriate sections before making your selection.

☒ Life sciences ☐ Behavioural & social sciences ☐ Ecological, evolutionary & environmental sciences

For a reference copy of the document with all sections, see nature.com/documents/nr-reporting-summary-flat.pdf

Life sciences study design

All studies must disclose on these points even when the disclosure is negative.

Sample size	Samples were gathered via colectomy of six patients that visited Stanford University Hospital between November 2018 and December 2020. Samples were selected that had sufficient material to assay per -omic assay type. We selected the maximum number of samples that we could afford to assay.
Data exclusions	For all -omics types, only samples from patients A001, A002, F, G, A014, and A015 were ultimately included for analysis as patient B001 samples were excluded on the basis that B001 did not have the cancer condition of interest. For the transcriptomics data, a RIN cutoff of 6.0 was applied that excluded a number of samples - this criteria was not pre-determined but rather adjusted based on the PCA spread of the data. For the other -omics, all such samples that passed pre-determined QC for the aforementioned six patients were included.
Replication	Replication was confined predominantly to experimental assays and quantification of molecular abundance for each -omic type. For the proteomics data, each sample was run with two replicates and their normalized abundance compared graphically via PCA. For the metabolomics and lipidomics data, three samples were replicated across two batches and their normalized abundances were checked graphically via PCA to disconfirm batch effects. For the transcriptomics data, technical replicates were confined to the third batch and the clustering of technical replicates normalized gene counts were checked graphically via PCA.
Randomization	We controlled for batch covariates in the differential analysis.
Blinding	There was no clinical intervention and therefore no blinding.

Reporting for specific materials, systems and methods

We require information from authors about some types of materials, experimental systems and methods used in many studies. Here, indicate whether each material, system or method listed is relevant to your study. If you are not sure if a list item applies to your research, read the appropriate section before selecting a response.

Materials & experimental systems

n/a	Involved in the study
☒	☐ Antibodies
☒	☐ Eukaryotic cell lines
☒	☐ Palaeontology and archaeology
☒	☐ Animals and other organisms
☒	☐ Clinical data
☒	☐ Dual use research of concern
☒	☐ Plants

Methods

n/a	Involved in the study
☒	☐ ChIP-seq
☒	☐ Flow cytometry
☒	☐ MRI-based neuroimaging

Plants

Seed stocks	*Report on the source of all seed stocks or other plant material used. If applicable, state the seed stock centre and catalogue number. If plant specimens were collected from the field, describe the collection location, date and sampling procedures.*
Novel plant genotypes	*Describe the methods by which all novel plant genotypes were produced. This includes those generated by transgenic approaches, gene editing, chemical/radiation-based mutagenesis and hybridization. For transgenic lines, describe the transformation method, the number of independent lines analyzed and the generation upon which experiments were performed. For gene-edited lines, describe the editor used, the endogenous sequence targeted for editing, the targeting guide RNA sequence (if applicable) and how the editor was applied.*
Authentication	*Describe any authentication procedures for each seed stock used or novel genotype generated. Describe any experiments used to assess the effect of a mutation and, where applicable, how potential secondary effects (e.g. second site T-DNA insertions, mosiacism, off-target gene editing) were examined.*
